# Phylogenetic and evolutionary analysis of VP1 coding sequences of foot-and-mouth disease virus serotypes A, O, and SAT2 in Egypt

**DOI:** 10.1186/s12985-025-03039-4

**Published:** 2025-12-27

**Authors:** Alyaa Elrashedy, Mohamed Nayel, Akram Salama, Ahmed Zaghawa, Ahmed Badr, Mohamed E. Hasan

**Affiliations:** 1 Department of Animal Medicine and Infectious Diseases (Infectious Diseases), Faculty of Veterinary Medicine, University of Sadat City, Sadat City, Egypt; 2 Bioinformatics Department, Faculty of Biotechnology, University of Sadat City, Sadat City, Egypt

**Keywords:** Foot and mouth disease, Phylogenetic, Multiple sequence alignment, VP1, Vaccine, Egypt

## Abstract

**Background:**

Foot-and-mouth disease (FMD) is a highly infectious viral disease caused by the foot-and-mouth disease virus (FMDV), which has seven serotypes requiring serotype-specific vaccines due to the absence of cross-protection. Understanding the genetic evolution of circulating strains is crucial for effective disease control and vaccine design. This study provides the first comprehensive evolutionary analysis of FMDV serotypes A, O, and SAT2 circulating in Egypt (1972–2022), integrating molecular clock modeling and structural analysis to uncover recent viral diversification. This study focused on analyzing the viral protein 1 (VP1) coding sequences from Egyptian field strains of serotypes A, O, and SAT2 using in silico approaches.

**Methods:**

The nucleotide and amino acid sequences of VP1 for FMDV serotypes A, O, and SAT2 circulating in Egypt were retrieved from GenBank. Multiple sequence alignment was performed using ClustalW in MEGA 11, followed by phylogenetic tree construction using the maximum likelihood method with 1,000 bootstrap replicates. Pairwise identity matrices were generated to assess nucleotide similarities among isolates. Time-calibrated phylogenetic analyses were conducted using BEAST v2.6 to estimate substitution rates per site per year. Mutational analysis of the VP1 protein, particularly within the RGD (Arg-Gly-Asp) motif, was performed to identify novel amino acid substitutions with potential functional significance.

**Results:**

Multiple sequence alignment, phylogenetic analysis, and identity matrix comparisons were conducted to evaluate genetic relationships, while time-calibrated phylogenetic analysis estimated substitution rates per site per year. Serotype A strains clustered within Asia and Africa topotypes, with the first detection of a novel Europe–South America (Europe-SA) topotype in Egypt. Serotype O strains were grouped into East Africa (EA-3), Middle East–South Asia (ME-SA), and another new Euro–SA topotype was identified in Sharqia Governorate. Serotype SAT2 strains consistently aligned with topotype VII, with clustering patterns noted in 2012 and 2018 isolates. Importantly, a novel G136S mutation was identified within the conserved RGD motif of the Menoufia strain (MG552839), representing the first report of this substitution. The estimated mean evolutionary rates were 2.23 × 10⁻³, 1.85 × 10⁻³, and 4.48 × 10⁻⁶ substitutions per site per year for serotypes A, O, and SAT2, respectively.

**Conclusions:**

This study extends previous molecular investigations by including recent isolates from nearly all Egyptian governorates and integrating quantitative evolutionary rate estimation with structural analysis. The detection of new topotypes and unique mutations provides novel insights into FMDV evolution in Egypt and emphasizes the need for continuous molecular surveillance and periodic vaccine updates to maintain protection against emerging lineages.

**Supplementary Information:**

The online version contains supplementary material available at 10.1186/s12985-025-03039-4.

## Background

Foot-and-mouth disease (FMD) is an economically important viral disease that affects domestic and wild cloven-hooved animals [1]. The disease is caused by the Foot and Mouth Disease Virus (FMDV), a virus belonging to the *Picornaviridae* family and the *Aphthovirus* genus [2]. Primary modes of FMD transmission involve direct or indirect interaction with diseased animals, their infected secretions, such as saliva, nasal discharges, and feces, as well as contaminated materials like food, vehicles, and equipment that have come into contact with these discharges. FMD-induced clinical symptoms comprise fever, extensive vesicular lesions on the mouth, nose, limbs, and udders, along with indications such as lameness, declined milk production, and a deterioration in overall health status [3, 4]. FMDV has seven distinguishing immunological serotypes, including A, O, C, Asia, and Southern African territories (SAT1-3), exhibiting over 65 genetic lineages and topotypes [5]. Topotypes are determined based on specific genetic characteristics and the geographical distribution of viral strains, often reflecting the evolutionary history and adaptation of the virus across various regions. In contrast, genetic lineages refer to the genetic variations within a specific serotype, highlighting the evolutionary relationships among strains [6]. While both terms relate to the classification of viral strains, topotypes focus on geographical and phenotypic traits, while genetic lineages emphasize genetic similarities and differences [7, 8].

In Egypt, serotypes A, O, and SAT2 are recognized as the causative agents of FMD. The level of divergence among these serotypes is significant, with each serotype exhibiting distinct genetic and immunological properties. This divergence complicates vaccine development and efficacy, as vaccines targeting one serotype may not provide adequate protection against others [9]. These three serotypes are analyzed separately due to their unique epidemiological characteristics and the distinct lineages that circulate within each serotype [10]. Additionally, while they can be aligned for comparative analysis, the degree of genetic variability may affect the alignment’s quality, making it essential to consider their differences during analysis. The continuous introduction of new lineages from external sources creates an unstable environment, making it difficult to select appropriate vaccination antigens. One of the primary reasons for the prevalence of FMD in Egypt is the unrestricted movement of animals and the importation of livestock [11].

Mass vaccination is regarded as the predominant approach for FMD management; however, the current use of inactivated vaccines poses a formidable challenge due to their lack of cross-protective efficacy [12]. This limitation affects not only the ability to protect the seven distinct serotypes of FMDV but also within individual serotypes, making it difficult to control outbreaks effectively [13]. As a result, outbreaks can occur even in vaccinated populations, undermining control efforts and leading to economic losses in livestock industries. Effective vaccination strategies are crucial for preventing disease spread, and the inability of inactivated vaccines to offer broad protection complicates these efforts [14, 15]. This predicament is exacerbated by the pronounced genetic variability during RNA replication, coupled with substantial inter- and intra-serotype recombination [16].

Advancements in bioinformatics have significantly enhanced our ability to study the genetic diversity and evolution of FMDV, providing valuable insights for vaccine development and disease control [17]. Multiple sequence alignment (MSA) enables the identification of conserved and variable regions across different viral protein 1 (VP1) sequences, shedding light on genetic stability and divergence. Phylogenetic analysis helps reconstruct the evolutionary relationships among circulating strains, offering a clearer picture of lineage emergence and viral transmission patterns [18, 19]. Sequence similarity analysis assesses genetic distances between strains, which is essential for understanding antigenic variation and potential vaccine mismatches. Molecular clock modeling estimates mutation rates and the timescale of viral evolution, providing a deeper understanding of FMDV’s adaptive mechanisms [20]. Additionally, three-dimensional (3-D) structure prediction of VP1 reveals potential structural changes that may affect antigenicity, while mutation analysis identifies key amino acid substitutions that could influence viral fitness, immune escape, and vaccine efficacy [21]. Integrating these bioinformatics approaches enhances our ability to monitor viral evolution and optimize vaccine strategies for effective FMDV control.

Recent studies have provided valuable insights into the phylogenetic clustering and epidemiology of FMDV serotypes circulating in Egypt [7, 22, 23]. However, a detailed quantitative analysis of their evolutionary dynamics and the structural impact of novel mutations remains limited. To address this gap, the present study has three main objectives. First, it applies a robust Bayesian molecular clock approach to estimate substitution rates for serotypes A, O, and SAT2. Second, it conducts a comprehensive mutational analysis of the VP1 protein, which plays a crucial role in eliciting the immune response and is the key determinant of viral serotype and pathogenicity [24]. Third, it integrates in silico structural modeling to evaluate the potential functional effects of key amino acid changes.

This work also incorporates a broader and more up-to-date field strains than previous reports, including isolates collected from nearly all Egyptian governorates available in GenBank, providing extensive temporal and spatial coverage. By combining evolutionary and structural analyses, this study offers an updated and comprehensive understanding of the molecular evolution and circulating status of FMDV in Egypt, with implications for vaccine efficacy and strain selection.

## Materials and methods

### Data retrieval

The complete VP1 nucleotide sequences of FMDV serotypes A, O, and SAT2 from Egyptian field strains were retrieved from the National Center for Biotechnology Information (NCBI) database (Table S1). In total, 59 sequences were available for serotype A, 50 for serotype O, and 50 for serotype SAT2. To avoid redundancy and ensure a representative dataset, identical sequences were identified and removed. After this filtering step, the final dataset included 29 unique sequences for serotype A, 35 for serotype O, and 24 for serotype SAT2. This non-redundant dataset was then used for phylogenetic and evolutionary analyses.

### Multiple sequence alignment

The deduced protein of the Egyptian serotypes A, O, and SAT2 of FMD from their nucleotide sequences was multiple aligned using CLUSTAL W of BioEdit software. The CLUSTAL W enhances the sensitivity of progressive Multiple Sequence Alignment (MSA) through sequence weighting, position-specific gap penalties, and weight matrix selection [25].

### Sequence identity matrix

The Sequence Demarcation Tool Version 1.3 (SDTv1.3) is used to analyze the sequence pairwise identity matrix of the nucleotide sequences [26]. It aligned every unique pair of sequences (using the MUSCLE program [27]) and then presented the results in a colorful matrix for easy understanding. The similarity scores are calculated using the formula 1 - (M/N), where M is the number of mismatching nucleotides and N is the total number of positions in the alignment where neither sequence has a gap.

### Phylogenetic and molecular evolutionary analysis

For phylogenetic analyses, firstly, the best model selection was performed using the “find best DNA/protein models” option in MEGA 11, which informed the choice of model for phylogenetics [28]. The analysis was conducted on the nucleotide level using the Tamura-Nei model, which estimates the nucleotide substitutions using mathematical accounting for transitions and transversions, which are common in RNA viruses like FMDV due to their high mutation rates and selective pressures, excess transitions, unequal nucleotide frequencies, and site-specific variation while providing confidence intervals for evolutionary timelines [29]. The choice of this model ensured an accurate representation of evolutionary distances and clustering within the serotype-specific topotypes. Additionally, phylogenetic analysis was performed on the amino acid substitution types using the Jones-Taylor-Thornton (JTT) matrix-based model, which facilitates the creation of updated mutation data matrices from large protein sequence datasets by clustering sequences at 85% identity, aligning closely related pairs, and counting amino acid exchanges [30].

The phylogenetic analysis was performed on the nucleotide and amino acid levels using the Maximum Likelihood (ML) method [29] in MEGA11 software with the Tamura-Nei model and the JTT model, respectively, applying a bootstrap threshold of 70% from 1000 replicates to enhance the reliability of the confidence values assigned to each internal node of the phylogenetic tree [31].

Also, MEGA11 software was employed to estimate pairwise distances and the overall mean distance of the amino acid sequences using the JTT matrix-based model [30]. For pairwise distance calculations, the “compute pairwise distances” option in the distance menu of MEGA11 was utilized, with default settings adjusted only for the variance estimation method, which was set to the bootstrap method with 1000 replicates. The overall mean distance was calculated using the “compute overall mean distance” option from the same menu, maintaining default settings.

The substitution matrix of the amino acid sequences was estimated using the “estimate substitution matrix” option in the model menu of MEGA 11 with the JTT model. The transition/transversion bias was assessed for the nucleotide sequences with the Tamura-Nei model.

### Temporal signal analysis

Before performing molecular clock analysis, the presence of a temporal signal in the dataset was assessed using root-to-tip regression analysis in TempEst software [32]. The root-to-tip distances were plotted against the sampling dates of the sequences, and the correlation coefficient (R²) was calculated to evaluate the strength of the temporal signal. A significant positive correlation (*p* < 0.05) was considered evidence of sufficient temporal signal to proceed with molecular clock analysis.

### Molecular clock analysis

Time-resolved phylogenetic trees for the three serotypes (A, O, and SAT2) were estimated using the BEAST.X (v10.5.0-beta5) software package [33]. The analysis utilized the Tamura-Nei model of nucleotide sequence evolution, incorporating gamma-distributed rate variation and invariant sites across four categories. The selection of the Tamura-Nei model was based on Bayesian Information Criteria (BIC) results obtained from a statistical evaluation using MEGA11 software to identify the best nucleotide substitution model. A lognormal uncorrelated relaxed clock model was used for the analysis [34], and the coalescent constant size model was set as the prior tree model [35]. The Markov Chain Monte Carlo (MCMC) [36] process was run for 500 million generation steps, sampling trees every 50,000 steps after a burn-in period of 10% of the chain. The estimates yielded an effective sample size (ESS) greater than 200. The Tracer software was used to analyze the substitution models, clock models, and effective population size tree prior models [37].

### Tertiary structure prediction of the VP1 protein

The VP1 protein was retrieved from the UniProtKB database with an accession number (P03305). The tertiary structure (3-D) prediction was conducted using the alphafold2 server. It is an advanced artificial intelligence (AI) system created by DeepMind that predicts the 3D structures of proteins from their amino acid sequences with remarkable accuracy [38]. Subsequent release of structures of more than 200 million proteins predicted by the alphafold2 further aroused great enthusiasm in the scientific community, especially in the fields of biology and medicine. The 3D predicted protein was visualized using Jmol and Discovery Studio version 2021, with the presentation of the highlighted mutations at the significant residues.

## Results

### Multiple sequence alignment

VP1 protein is the most important antigenic protein of FMDV, given to the G-H loop, antigenic sites, and other biologically important domains. The N-terminal (1–30) of the VP1 protein has an essential role in the overall structural stability of the viral capsid. The C-terminal region (positions 190–214) is involved in viral assembly and packaging, contributing to capsid stability. The B-C loop (positions 40–60) and the E-F loop (positions 70–90) are often involved in antigenic sites and play a significant role in receptor binding and immune recognition [39, 40]. However, the G-H loop (130–160) is a key functional region involved in receptor binding (integrins) and is a major target for neutralizing antibodies, as it contains a highly conserved Arg-Gly-Asp (RGD) motif [41]. Mutations in these regions can significantly affect viral infectivity and immune escape. The multiple sequence alignment of the three serotypes (A, O, and SAT2) in Egypt has been conducted and showed conservation and non-conservation mutations in these regions between the field and vaccinal strains, which explain the status of the FMD in Egypt.

**Serotype A**

**Fig. 1:**
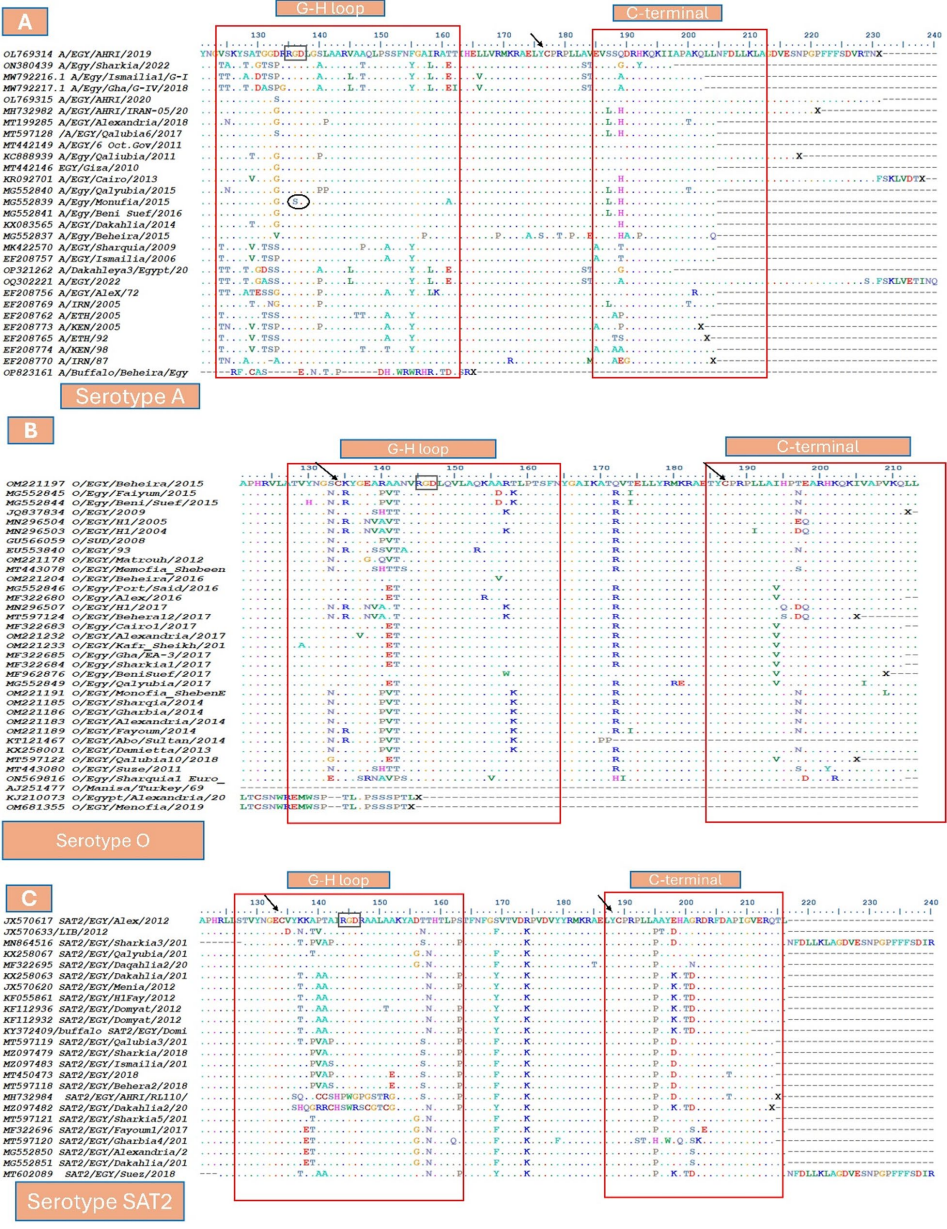
Multiple sequence alignment of VP1 protein of serotypes A (**A**), O (**B**), SAT2 (**C**) of FMDV in Egypt. Red boxes showed the antigenic sites of the G-H loop and C-terminal, the black boxes referred to the RGD motif in the three serotypes, and the black arrow pointed to the Cysteine conserved residues in the different positions for the three serotypes. In serotype A, the black circle is indicated to induce a mutation in the motif “G136S” in the MG552839 Menoufia

A comparison of the amino acid composition of VP1 from Egyptian field strains of FMDV serotype A was conducted. Comparing (OL769314 AHRI/2019) strains with the circulating field strains reported similarity and diversity in the antigenic sites. The N- and C-terminals of the VP1 protein revealed conservation. In the C-terminus, the conserved cysteine residue (C177) has a role in protein structure stability through disulfide bond formation. The B-C loop exhibited conservation, except for the mutations A51G, F56L, and A59S (Figure S1). The E-F loop displayed conserved antigenic sites, except for the substitution of lysine (K) at position 74 with different amino acids: glutamic acid (E), glutamine (Q), and aspartic acid (D). The G-H loop variations shown in **(**Fig. 1A**)**, are among the most variable, impacting the efficacy and matching of vaccines. It is noted that the RGD motif is conserved at 135–137 positions in all strains except G136S in MG552839 Menoufia. In contrast, positions 170–180 showed high conservation. Interestingly, it was noted that there are strains from the same year that have differences in their sequences. For example, in 2018, the strains from Ismailia (MW792216) and Gharbia (MW792217) exhibited N34S and L90S mutations.

**Serotype O**

The MSA of VP1 of (OM221197/Beheira/2015) and Egyptian field strains of FMDV serotype O was performed. The MSA revealed high conservation in the N-terminal region, with exceptions for the mutations V21E and V24I or V24D. The B-C loop showed conservation, except for positions Q45K, S46D, S46P, S46A, T48I, and S58A or S58P (Figure S2). The E-F loop exhibited complete conservation. However, the G-H loop contains variable mutations; it maintains a fixed position for the RGD motif at 145–147 positions. The region from positions 170 to 180 shows high conservation, with exceptions at Q172R and T174I. The C-terminal region is completely conserved, except for variations at position T197, where substitutions include S, E, N, or D. It is noteworthy that the Cysteine residue is conserved in different positions at the G-H loop (C134) and at the C-terminus (C187), which gives Serotype O more stability than Serotype A. What’s more, ON569816_Sharkia_Euro_South_America (the novel topotype) has + 6 mutations over the others L36M, A74S, V78I, N85D, A155V, and K202R (Fig. 1B).

**Serotype SAT2**

The MSA was executed on the VP1 amino acid sequence of FMDV serotype SAT2 field strains from 2012 to 2019 in Egypt. The (JX570617 EGY/2012) was set as the reference protein for comparison. Serotype SAT2 was revealed as the most stable, other than serotypes A and O. Given that it includes four conserved cysteine residues at 74, 80 (in E-F loop), 134 (in G-H loop), and 189 (in C-terminus), which enhance the disulfide bond in the protein structure. It is shown that they are conserved in the N-terminal and mainly conserved in the C-terminal except for A195P, E198D or E198K, A200T, and G201D (Figure S3). The B-C loop exhibited changes in N45G, T48S, D55N, K57R, and K58E. The E-F loop has substitutions at D83E and A88V. The region from (100–130) has complete conservation. The G-H loop also showed variation (Fig. 1C). The RGD motif is conserved at the 144–146 position in all strains except the conserved patterns “PWG” and “SWR”, which appeared in MH732984_AHRI/2018 and MZ097482_Dakahlia2/2018 strains, respectively. Interestingly, KF112932, KF112936, and KY372409 (isolated from buffalo) field strains from the same location (Domyat) and in the same year 2012 have mutations between each other (Table 1).

**Table 1:** Summary of VP1 mutations, evolutionary rates, and unique findings for FMDV serotypes A, O, and SAT2 in Egypt

Serotype	Topotypes detected	Key mutations (all reported)	Substitution rate (subs/site/year)	Unique findings
**A**	Asia, Africa, Europe-SA	B-C loop: N34S, A51G, F56L, A59S. E-F loop: K74E/Q/D, L90S G-H loop: G136S (RGD motif)	2.23 × 10⁻³	First report of G136S mutation in conserved RGD motif; differences noted between strains from the same year (2018)
**O**	EA-3, ME-SA, Europe-SA	N- terminal: V21E, V24I/D. B-C loop: L36M, Q45K, S46D/P/A, T48I, S58A/P. E-F loop: Q172R, T174I, A74S, V78I, N85D C-terminal: T197S/E/N/D, A155V, K202R.	1.85 × 10⁻³	New Euro-SA lineage detected in 2022; high structural stability due to conserved cysteines; multiple loop mutations affecting antigenicity
**SAT2**	VII (2012–2019)	B-C loop: N45G, T48S, D55N, K57R, K58E. E-F loop: D83E, A88V. C-terminal: A195P, E198D/K, A200T, G201D	4.48 × 10⁻⁶	Stable topotype VII circulation; buffalo-associated variants; unique PWG/SWR motifs observed in 2018 strains

### 3.2. Sequences identity matrix

**Fig. 2:**
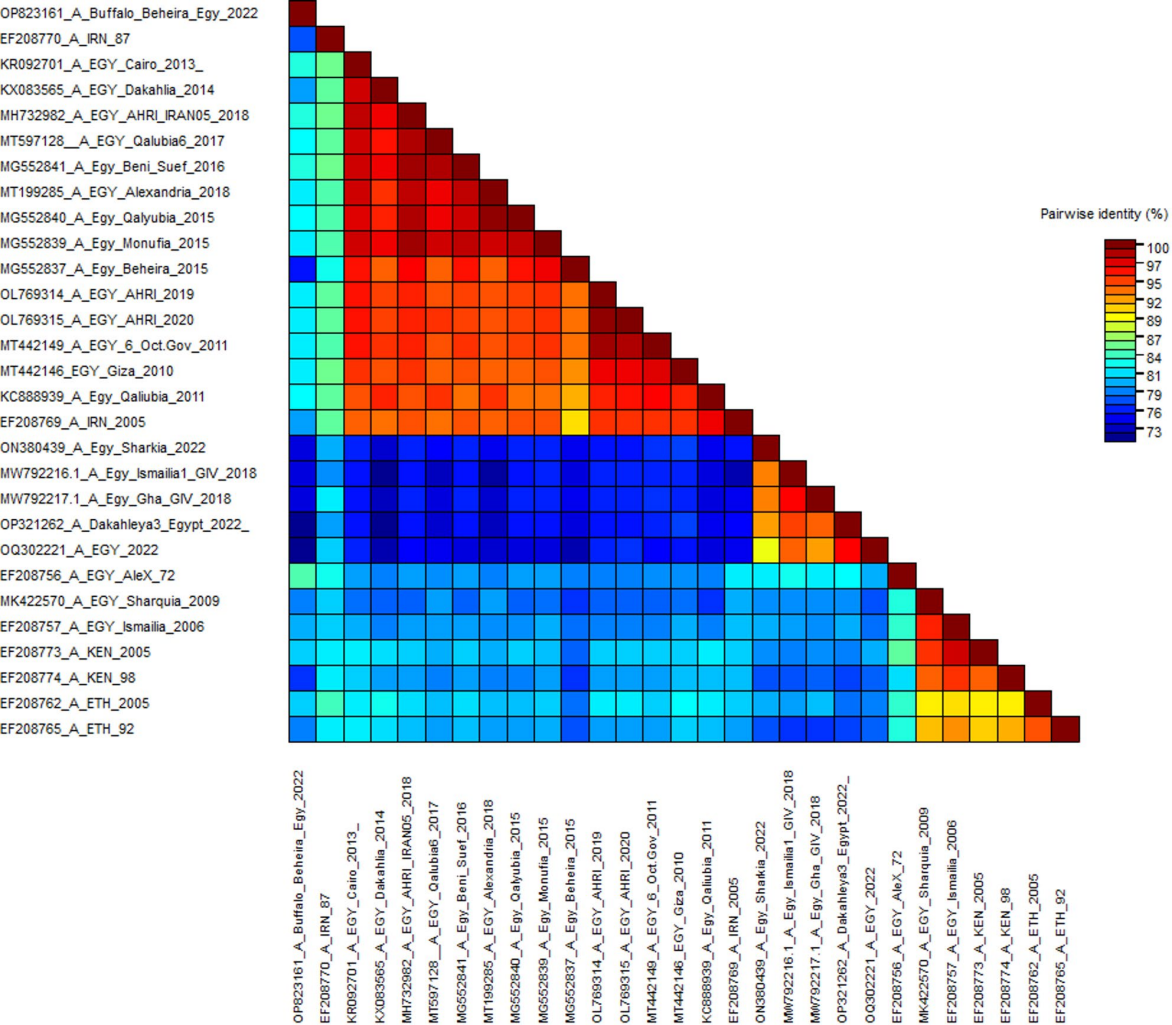
The pairwise percentage’s identity of the VP1 coding sequence of FMDV serotype A is shown in a plot generated by the Sequence Demarcation Tool (SDT) software. The analysis was performed between the field isolates. Sequences were aligned using the MUSCLE algorithm, and identity values were displayed as the ligand described

**Fig. 3:**
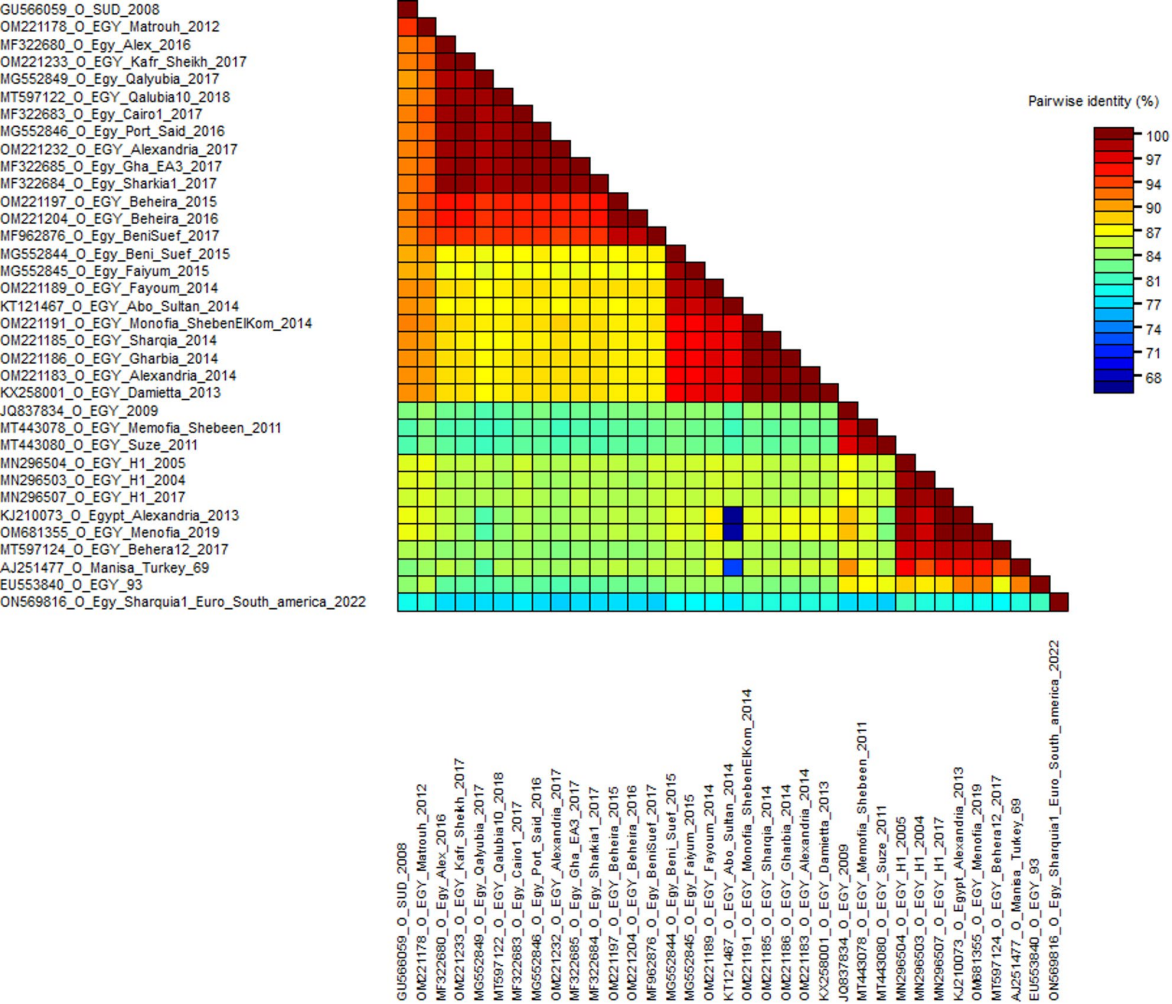
The pairwise percentage’s identity of the VP1 coding sequence of FMDV serotype O is shown in a plot generated by the Sequence Demarcation Tool (SDT) software. The analysis was performed between the field isolates. Sequences were aligned using the MUSCLE algorithm, and identity values were displayed as the ligand described

**Fig. 4:**
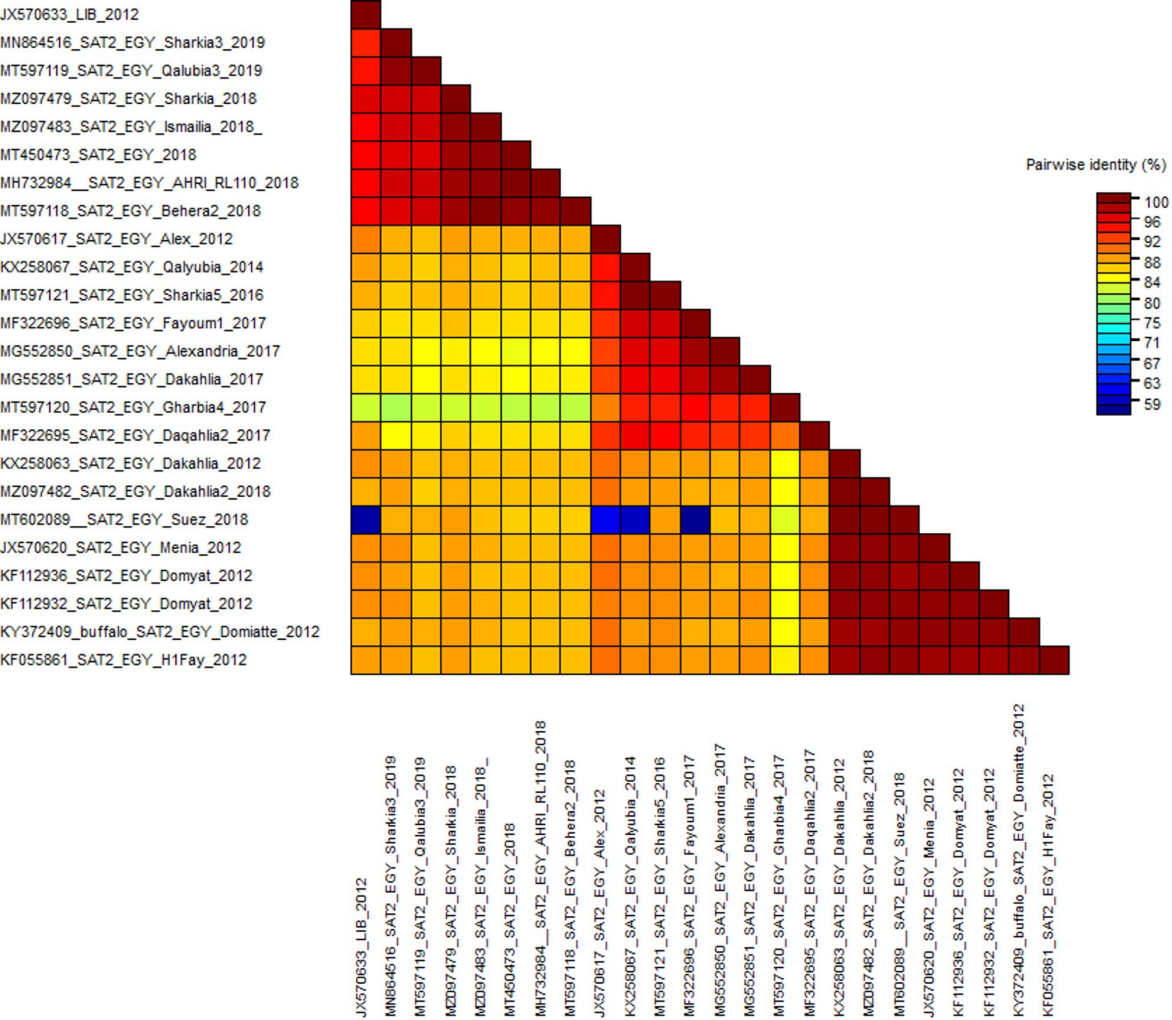
The pairwise percentage’s identity of the VP1 coding sequence of FMDV serotype SAT2 is shown in a plot generated by the Sequence Demarcation Tool (SDT) software. The analysis was performed between the field isolates. Sequences were aligned using the MUSCLE algorithm, and identity values were displayed as the ligand described

According to the MSA of the three FMD serotypes A, O, and SAT2, these sequences’ identity percentages were calculated using the sequence demarcation tool. The nucleotide sequence identity percentage between the circulating field isolates ranged from 73 to 100%, 68 to 100%, and 59 to 100% for serotypes A, O, and SAT2, respectively (Figs. 2, 3 and 4).

### 3.3. Phylogenetic analysis

Based on the lowest BIC scores (Bayesian Information Criterion), the Tamura-Nei model was found to be the best model for describing the substitution pattern of the nucleotide sequences, and the JTT matrix-based model was the best for the amino acid sequences. The evolutionary history was inferred using the Maximum Likelihood method with the two best models for the DNA and protein sequences for the three serotypes (A, O, and SAT2).

**Fig. 5:**
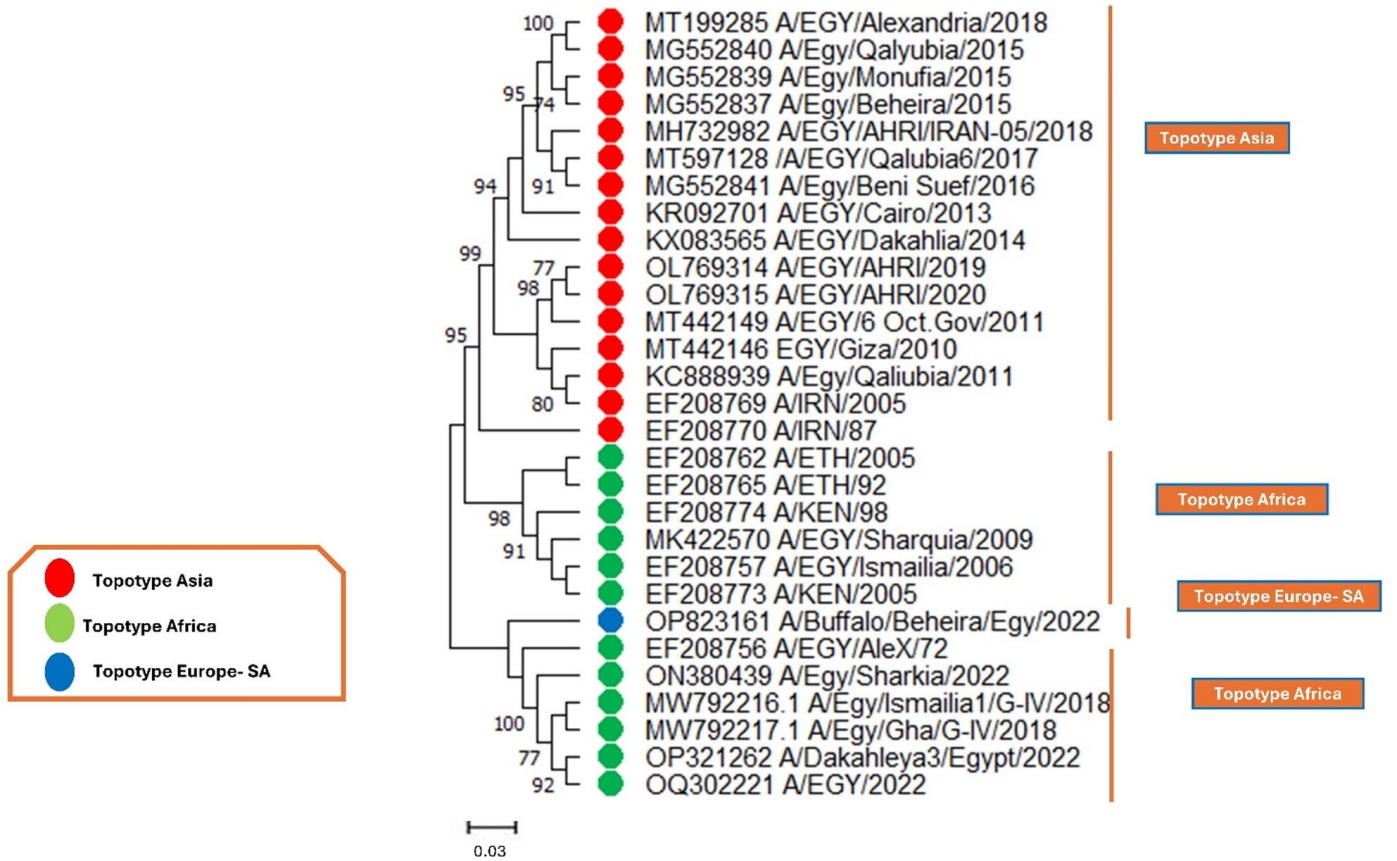
The evolutionary history was reconstructed using the Maximum Likelihood approach and the Tamura-Nei model for serotype A. The tree with the highest log likelihood value of −4069.36 is presented. The branches are annotated with the percentage of trees in which the associated taxa are grouped. Initial trees for the heuristic search were generated automatically using the Neighbor-Join and BioNJ algorithms applied to a pairwise distance matrix estimated with the Tamura-Nei model, followed by selecting the topology with the best log-likelihood. This analysis included 29 nucleotide sequences of serotype A, considering codon positions 1 st, 2nd, 3rd, and noncoding regions. The final dataset comprised a total of 770 positions. The red circles pointed to Asia, the green circles referred to Africa, and the blue circles indicated Europe-SA

In serotype A, two trees were conducted at the nucleotide (Fig. 5) and protein levels (Figure S4), clustering sequences into three distinct topotypes: Asia, Africa, and Europe-SA, spanning. The phylogenetic analysis revealed that the majority of Egyptian isolates belong to the Asia topotype (red), with strains collected between 2011 and 2019 closely related to those from Iran, highlighting potential transboundary virus introductions. The Africa topotype (green) includes historical and recent strains from Ethiopia, Kenya, and Egypt, suggesting ongoing viral circulation within the region. Notably, a few Egyptian strains from 2022 clustered within the Europe-SA topotype (blue), indicating potential genetic exchanges with European or South American lineages. The high bootstrap values support the robustness of these groupings, reflecting distinct evolutionary pathways among the topotypes. These findings provide crucial insights into the genetic diversity and evolutionary trends of serotype A in Egypt, which is essential for vaccine matching and disease control strategies. Also, this was convinced by the phylogenetic tree based on the protein sequences (Figure S4).

**Fig. 6:**
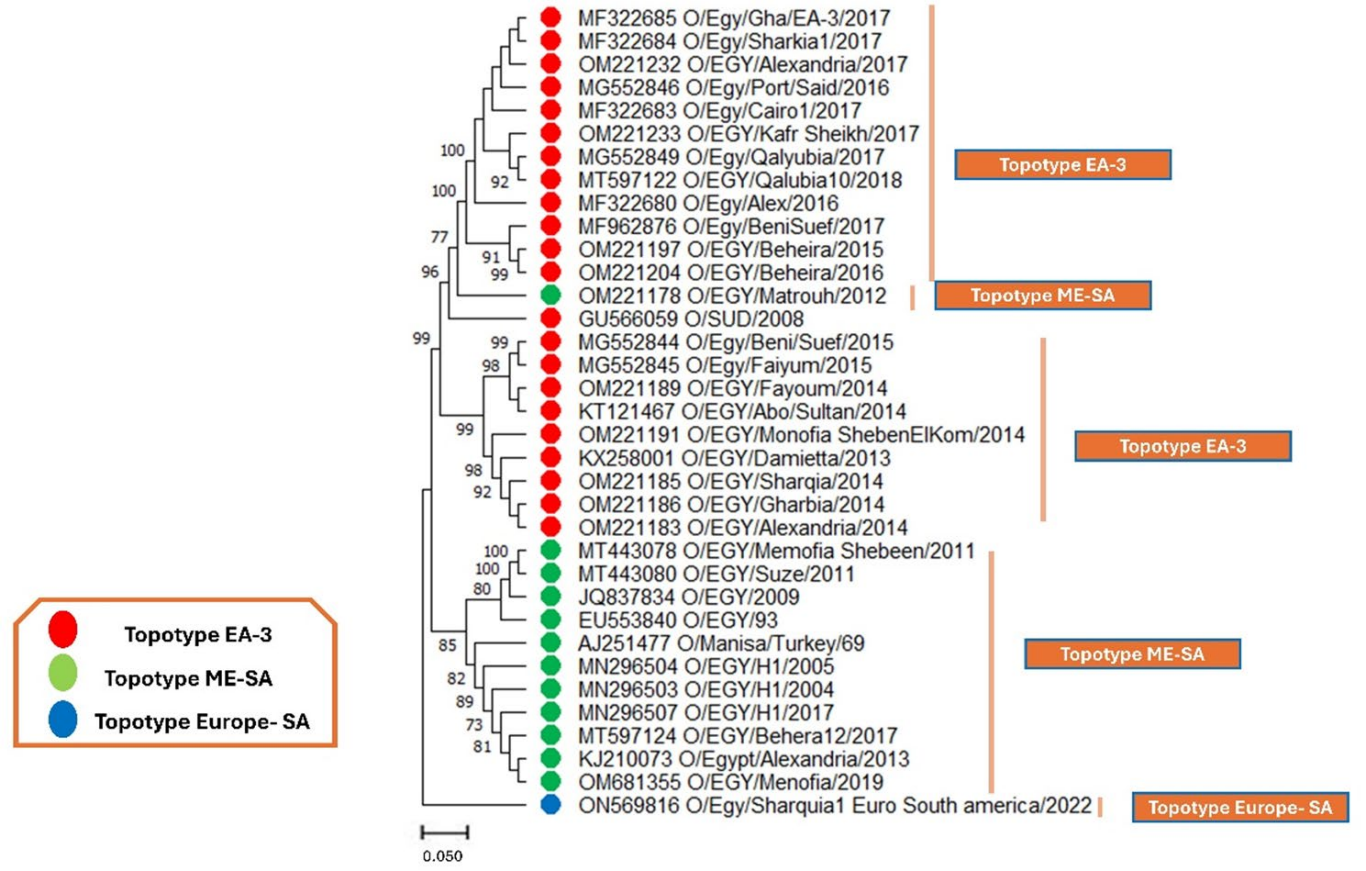
The evolutionary history was reconstructed using the Maximum Likelihood approach and the Tamura-Nei model for serotype O. The tree with the highest log likelihood value of −3579.64 is presented. The branches are annotated with the percentage of trees in which the associated taxa are grouped. Initial trees for the heuristic search were generated automatically using the Neighbor-Join and BioNJ algorithms applied to a pairwise distance matrix estimated with the Tamura-Nei model, followed by selecting the topology with the best log-likelihood. This analysis included 35 nucleotide sequences of serotype O, considering codon positions 1 st, 2nd, 3rd, and noncoding regions. The final dataset comprised a total of 710 positions. The red circles pointed to East Africa-3 (EA-3), the green circles referred to the Middle East-South Asia (ME-SA), and the blue circles indicated Europe-SA

On the other hand, the evolutionary phylogenetic trees of serotype O, at both the nucleotide (Fig. 6) and protein levels (Figure S5), also revealed three distinct clades: East Africa-3 (EA-3), Middle East-South Asia (ME-SA), and Euro-South America (Europe-SA). From 2013 to 2018, most field strains clustered into the EA-3 topotype (red), indicating the predominant circulation of this lineage in Egypt during that period. However, other isolates, primarily from 2004 to 2019, were grouped within the ME-SA topotype (green), suggesting a co-circulation of multiple lineages with possible introductions from neighboring regions. Notably, a recent 2022 isolate clustered within the Europe-SA topotype (blue), pointing to a potential incursion of a genetically distinct strain into Egypt. The high bootstrap values confirm the reliability of these groupings, reflecting the dynamic evolution of serotype O in the region.

**Fig. 7:**
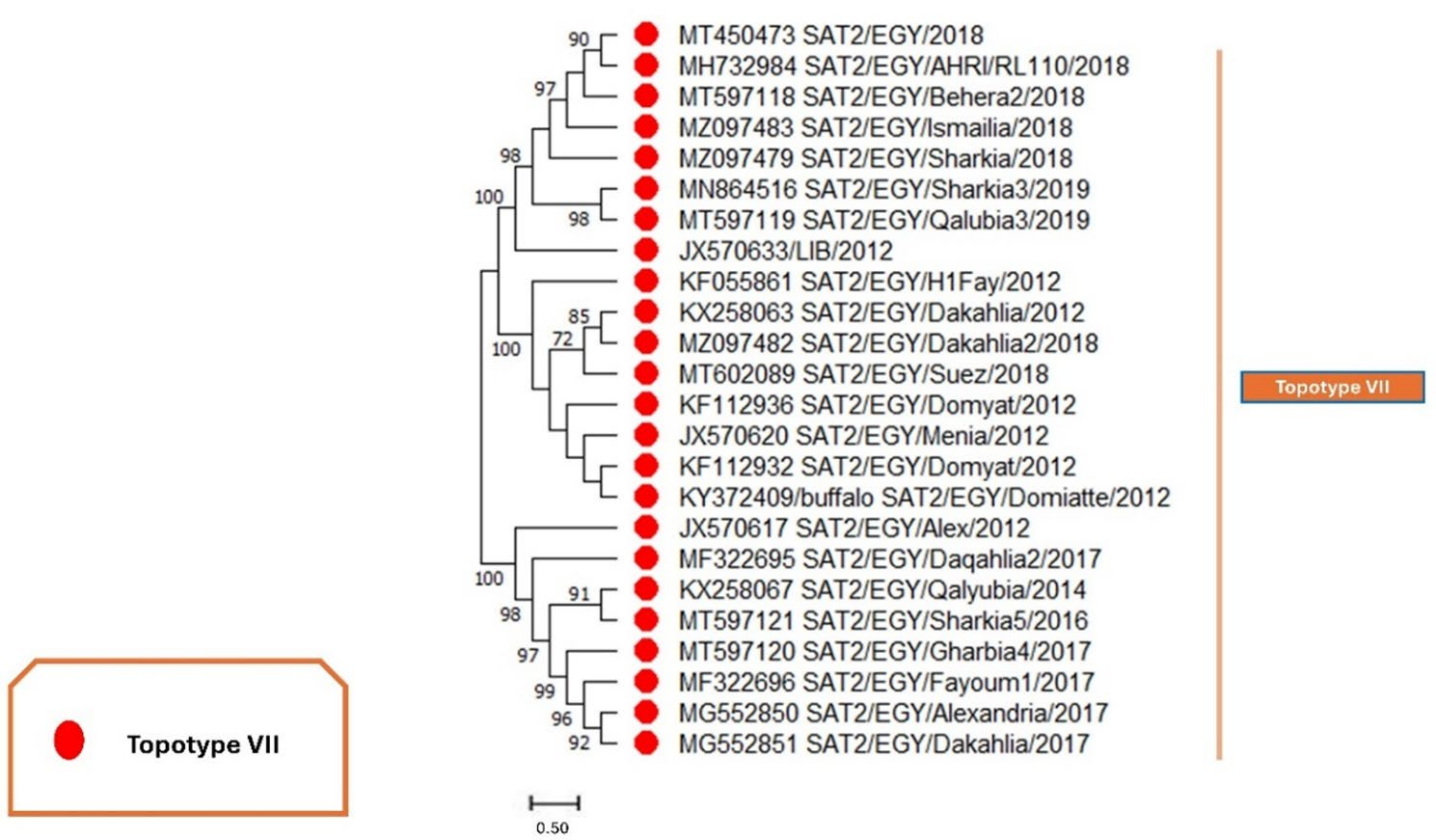
The evolutionary history was reconstructed using the Maximum Likelihood approach and the Tamura-Nei model for serotype SAT2. The tree with the highest log likelihood value of −3333.30 is presented. The branches are annotated with the percentage of trees in which the associated taxa are grouped. Initial trees for the heuristic search were generated automatically using the Neighbor-Join and BioNJ algorithms applied to a pairwise distance matrix estimated with the Tamura-Nei model, followed by selecting the topology with the best log-likelihood. This analysis included 24 nucleotide sequences of serotype SAT2, considering codon positions 1 st, 2nd, 3rd, and noncoding regions. The final dataset comprised a total of 1279 positions. The red circles pointed to topotype VII

Furthermore, in serotype SAT2, the phylogenetic trees based on nucleotide sequences (Fig. 7) and protein sequences (Figure S6) from 2012 to 2019 revealed a single topotype, VII. All circulating strains during this period were clustered within the VII topotype (red), with strong bootstrap support (Fig. 7). These findings highlight the persistence of the VII topotype in Egypt and emphasize the importance of continuous surveillance to detect potential new introductions or genetic shifts that may impact vaccine efficacy.

### Molecular evolutionary analysis

#### Maximum likelihood estimate of substitution matrix

The Jones-Taylor-Thornton (JTT) (1992) model was utilized to calculate substitution patterns and rates, where each element in the model reflects the probability of substitution (r) from one amino acid to another (row to column). When analyzing them, it is important to examine the relative values of instant r. The r values is made equal to 100, the amino acid frequencies are 7.69% (A), 5.11% (R), 4.25% (N), 5.13% (D), 2.03% (C), 4.11% (Q), 6.18% (E), 7.47% (G), 2.30% (H), 5.26% (I), 9.11% (L), 5.95% (K), 2.34% (M), 4.05% (F), 5.05% (P), 6.82% (S), 5.85% (T), 1.43% (W), 3.23% (Y), and 6.64% (V). This result was the same in the three serotypes (Table 2).

**Table 2:** Maximum Likelihood Estimate of Substitution Matrix

	A	*R*	*N*	D	C	Q	E	G	H	I	L	K	M	F	*P*	S	T	W	Y	V
**A**	-	0.14	0.12	0.22	0.06	0.12	0.34	0.67	0.03	0.10	0.15	0.11	0.06	0.03	0.51	1.37	1.39	0.01	0.02	1.01
**R**	0.21	-	0.10	0.04	0.11	0.64	0.10	0.53	0.38	0.07	0.18	2.01	0.05	0.01	0.19	0.35	0.20	0.09	0.04	0.06
**N**	0.22	0.12	-	1.48	0.03	0.16	0.19	0.30	0.48	0.13	0.06	0.78	0.04	0.02	0.03	1.79	0.71	0.00	0.12	0.06
**D**	0.33	0.04	1.22	-	0.01	0.11	2.49	0.49	0.12	0.03	0.03	0.09	0.02	0.01	0.03	0.21	0.13	0.00	0.08	0.11
**C**	0.23	0.27	0.07	0.03	-	0.02	0.02	0.21	0.09	0.04	0.08	0.02	0.05	0.14	0.03	0.76	0.14	0.08	0.35	0.21
**Q**	0.22	0.80	0.17	0.14	0.01	-	1.09	0.09	0.68	0.02	0.33	0.91	0.06	0.01	0.42	0.19	0.16	0.01	0.04	0.06
**E**	0.43	0.08	0.13	2.07	0.01	0.73	-	0.43	0.03	0.03	0.05	0.53	0.02	0.01	0.05	0.11	0.10	0.01	0.01	0.16
**G**	0.69	0.36	0.17	0.34	0.06	0.05	0.36	-	0.02	0.01	0.03	0.08	0.02	0.01	0.05	0.66	0.10	0.04	0.01	0.16
**H**	0.09	0.85	0.89	0.27	0.08	1.21	0.08	0.08	-	0.05	0.26	0.16	0.04	0.10	0.30	0.26	0.14	0.01	0.98	0.04
**I**	0.14	0.06	0.11	0.03	0.02	0.02	0.04	0.02	0.02	-	1.10	0.06	0.59	0.16	0.03	0.14	0.77	0.01	0.05	3.28
**L**	0.12	0.10	0.03	0.02	0.02	0.15	0.03	0.03	0.06	0.64	-	0.05	0.47	0.53	0.28	0.21	0.08	0.04	0.04	0.61
**K**	0.15	1.73	0.56	0.08	0.01	0.63	0.55	0.10	0.06	0.06	0.07	-	0.08	0.01	0.06	0.17	0.29	0.01	0.01	0.04
**M**	0.19	0.11	0.07	0.05	0.04	0.10	0.06	0.05	0.04	1.32	1.82	0.19	-	0.09	0.04	0.10	0.64	0.01	0.03	1.05
**F**	0.06	0.02	0.02	0.01	0.07	0.01	0.01	0.02	0.05	0.21	1.18	0.01	0.05	-	0.04	0.33	0.04	0.04	0.92	0.20
**P**	0.78	0.19	0.03	0.03	0.01	0.34	0.06	0.08	0.14	0.03	0.50	0.07	0.02	0.03	-	0.99	0.36	0.01	0.02	0.07
**S**	1.55	0.27	1.12	0.16	0.23	0.12	0.10	0.73	0.09	0.11	0.28	0.15	0.03	0.20	0.73	-	1.45	0.02	0.11	0.14
**T**	1.83	0.17	0.52	0.11	0.05	0.11	0.11	0.12	0.06	0.70	0.13	0.30	0.26	0.03	0.31	1.69	-	0.01	0.03	0.39
**W**	0.03	0.33	0.01	0.02	0.12	0.04	0.04	0.21	0.02	0.04	0.25	0.03	0.02	0.11	0.02	0.11	0.02	-	0.13	0.08
**Y**	0.06	0.06	0.15	0.12	0.22	0.05	0.02	0.02	0.70	0.08	0.11	0.03	0.02	1.15	0.03	0.22	0.06	0.06	-	0.06
**V**	1.16	0.05	0.04	0.08	0.07	0.04	0.15	0.18	0.01	2.60	0.83	0.04	0.37	0.12	0.06	0.14	0.35	0.02	0.03	-

#### 3.4.2. Maximum likelihood estimate of Transition/Transversion bias

The estimated Transition/Transversion bias (*R*) is 2.39 in serotype A, 3.08 in serotype O, and 3.55 in serotype SAT2. Substitution patterns and rates were estimated under the Tamura-Nei model. Each of the nucleotide frequencies is equal to 25.00%. In the three serotypes, the transition value was higher than the transversion values. This indicates that the substitutions were conservative and preserved the overall protein structure.

#### 3.4.3. Maximum composite likelihood estimate of the pattern of nucleotide substitution

The analysis was done using the Tamura-Nei model and each entry shows the probability of substitution (r) from one base to another (row to column). Transversional substitution rates were given in *italics*, while transitional substitution rates were shown in bold. For simplicity, the summation of r values is set to 100. The nucleotide frequencies are 24.24% (A), 19.43% (T/U), 30.39% (C), and 25.94% (G) in serotype A (Table 3). While in serotype O they were 21.96% (A), 25.62% (T/U), 39.82% (C), and 12.59% (G) (Table 4). Additionally, 23.90% (A), 18.47% (T/U), 32.05% (C), and 25.57% (G) were in serotype SAT2 (Table 5).

**Table 3:** Maximum Composite Likelihood Estimate of the stitution of Serotype APattern of Nucleotide Sub

	A	T	C	G
**A**	-	*2.26*	*3.53*	**16.11**
**T**	*2.81*	-	**27.83**	*3.01*
**C**	*2.81*	**17.79**	-	*3.01*
**G**	**15.05**	*2.26*	*3.53*	-

Transversional substitution rates were given in italics, while transitional substitution rates were shown in bold

**Table 4:** Maximum Composite Likelihood Estimate of the Pattern of Nucleotide Substitution in serotype O

	A	T	C	G
**A**	-	*0.54*	*0.84*	**0**
**T**	*0.46*	-	**58.28**	*0.27*
**C**	*0.46*	**37.51**	-	*0.27*
**G**	**0**	*0.54*	*0.84*	-

Transversional substitution rates were given in italics, while transitional substitution rates were shown in bold

**Table 5:** Maximum Likelihood Estimate of Substitution Matrix of Serotype SAT2

	A	T	C	G
**A**	-	*1.45*	*2.52*	**5.39**
**T**	*1.88*	-	**46.84**	*2.01*
**C**	*1.88*	**27**	-	*2.01*
**G**	**5.04**	*1.45*	*2.52*	-

Transversional substitution rates were given in italics, while transitional substitution rates were shown in bold

#### Estimation of average evolutionary divergence over all sequence pairs

Analyses were conducted using the Tamura-Nei model in MEGA11. The number of nucleotide substitutions per site from averaging over all sequence pairs is revealed as 0.19 in serotype A, 0.12 in serotype O, and 0.13 in serotype SAT2.

### Temporal signal analysis

The root-to-tip regression analysis provides insights into the evolutionary dynamics of the three FMDV serotypes: A, O, and SAT2. For serotype A, the dataset spans 50 years, with a moderate correlation coefficient (*R* = 0.67) and an R² value of 0.4577, indicating that approximately 45.77% of the genetic divergence can be explained by sampling time. The estimated evolutionary rate is 1.406E-3 substitutions per site per year. However, the wide range for the x-intercept (1894.33) suggests that this serotype has been evolving for over a century (TMRCA). The residual mean square error is relatively low (3.219E-4), indicating a reasonable fit of the regression model.

**Fig. 8:**
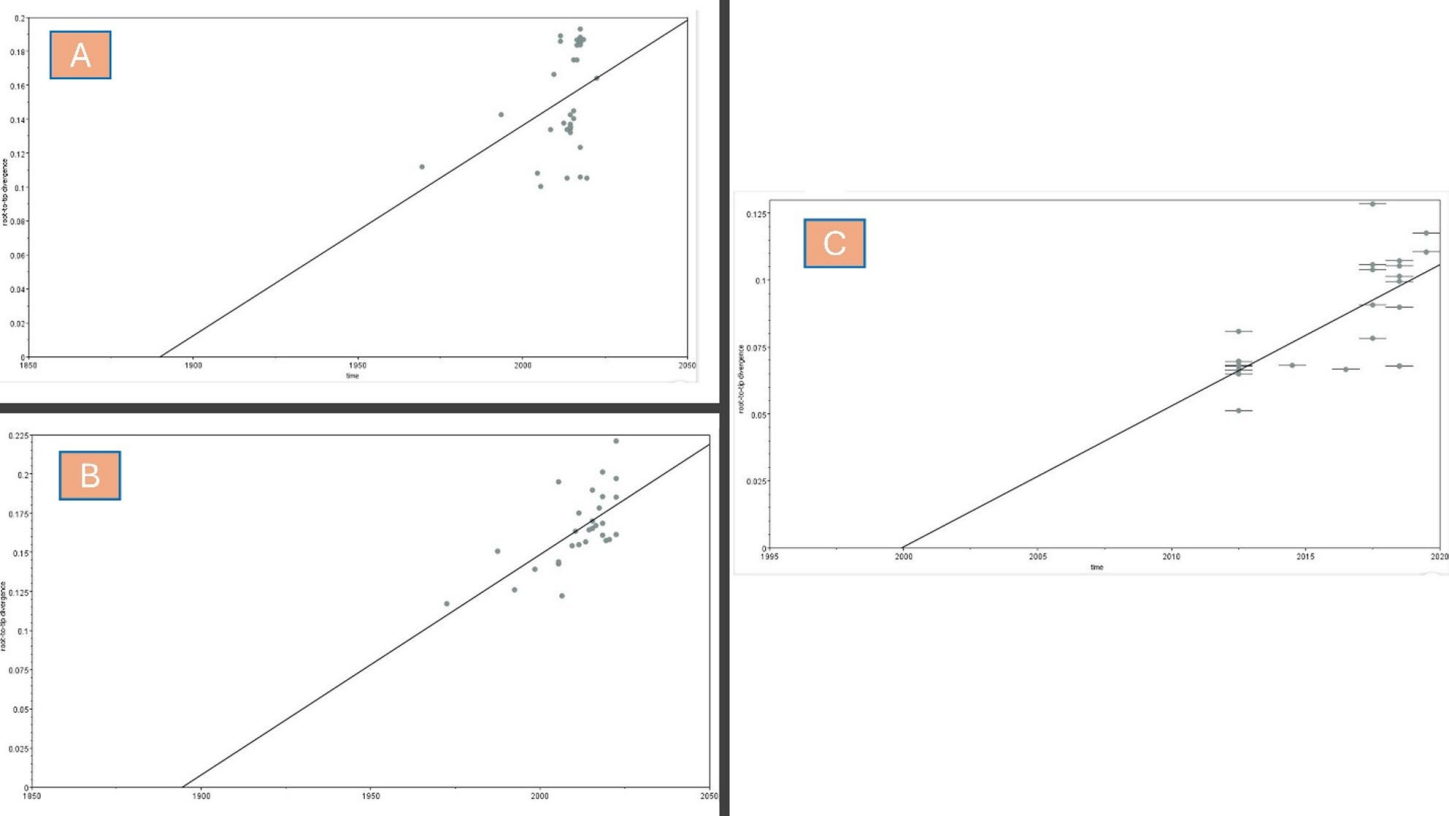
Root-to-tip regression analysis depicting the evolutionary dynamics of three FMDV serotypes: A, O, and SAT2. The slopes of the regression lines represent the evolutionary rates, measured in substitutions per site per year. Panel **A** shows serotype A with a moderate evolutionary rate of 1.406E-3. Panel **B** illustrates serotype O with a slightly lower rate of 1.239E-3, indicating slower genetic divergence. Panel **C** highlights serotype SAT2, which exhibits a significantly higher evolutionary rate of 5.2704E-3, suggesting rapid genetic change. The analysis provides insights into the distinct evolutionary patterns among the FMDV serotypes

However, for serotype O, the date range is 53 years; however, the temporal signal is weaker compared to serotype A. The correlation coefficient is 0.369, with an R² value of 0.136, indicating that only 13.6% of the genetic divergence is explained by sampling time. The evolutionary rate is slightly lower at 1.239E-3 substitutions per site per year. The TMRCA estimate is more precise (1890.07), but the higher residual mean square error (8.4215E-4) suggests greater variability in the data, which may affect the reliability of molecular clock analysis (Fig. 8).

Additionally, in serotype SAT2, the date range is much shorter at 7 years, yet it shows a moderate correlation coefficient (*R* = 0.7051) and an R² value of 0.4972. The evolutionary rate is higher at 5.2704E-3 substitutions per site per year, which could reflect faster evolutionary dynamics or limited sampling. The TMRCA estimate (1999.93) is more recent, and the residual mean square error is the lowest among the three serotypes (2.2935E-4), indicating a good fit of the regression model despite the shorter time frame.

### Molecular clock analysis

The molecular clock analysis of the three serotypes (A, O, and SAT2) OF FMDV using BEAST.X (v10.5.0-beta5) and Tracer software yielded important insights into the substitution rate and its uncertainty. The mean clock rate is estimated to be 2.23 × 10⁻³, 1.84 × 10⁻³, and 4.48 × 10⁻^6^ substitutions per site per year in the A, O, and SAT2 serotypes, respectively, indicating the average rate of evolution for the viral population.

### Tertiary structure prediction of VP1 protein

**Fig. 9:**
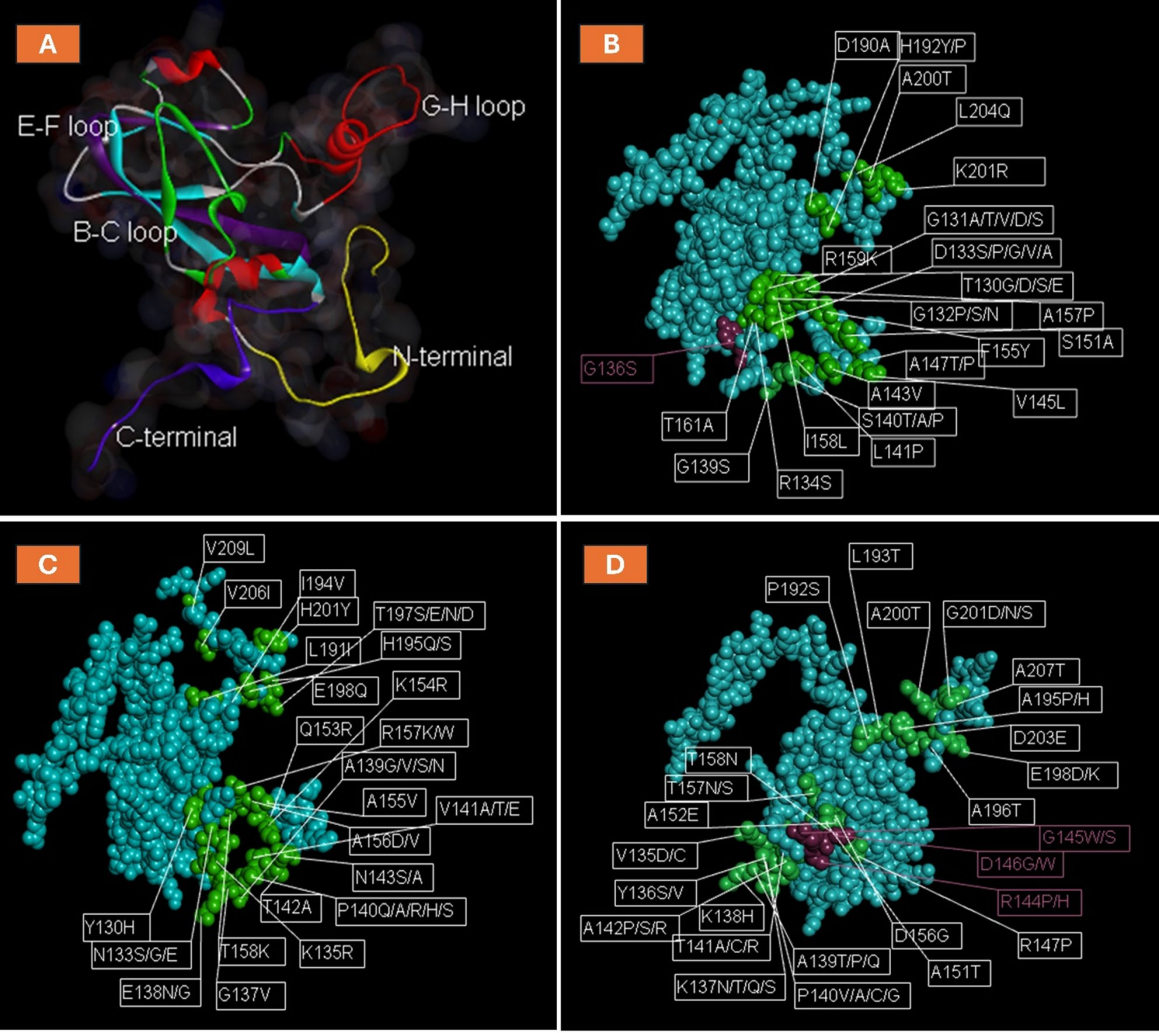
Cartoon diagram of the three-dimensional structure of viral protein 1 (VP1) of the FMDV using the Discovery Studio 2021 software for visualization (**A**). The N-terminal (1–30) was colored yellow; the B-C loop (40–60) was colored green, the E-F loop (70–90) was colored purple, the G-H loop (130–160) was colored red, and the C-terminal (190–214) was colored blue (**A**). The 3D structure of the VP1 protein highlighted the significant substitution in the G-H loop and C-terminal in serotype A (**B**), serotype O (**C**), and serotype SAT2 (**D**). The purple color mutation in (**B**, **D**) is related to a substitution occurring in the RGD motif

The VP1 protein plays a central role in FMDV antigenicity, particularly through its G-H loop, which harbors the RGD motif essential for integrin binding. Using AlphaFold2, the predicted 3D structure revealed critical regions such as the N-terminal (residues 1–30), G-H loop (130–160), and C-terminal (190–214), all contributing to structural stability and immune recognition (Fig. 9A). The analysis highlighted significant mutations in field strains that may impact VP1 function. For example, the G136S mutation within the conserved RGD motif in serotype A (Fig. 9B). Similarly, mutations like T197S and E198D in the C-terminal region in serotypes O and SAT2 (Fig. 9C and D), respectively. These findings underscore the importance of monitoring VP1 structural changes to ensure vaccine strains align with circulating variants, as mutations in key antigenic regions may diminish immune protection. Visualizations of these mutations further aid in understanding their impact on antigenicity, guiding improved vaccine formulations.

## Discussion

FMD is a contagious transmissible disease that has significant economic and trade implications during outbreaks. Despite efforts to reduce FMD through obligatory immunization, the disease remains dangerous in regions of Africa and Asia, given its high genetic variability and quasi-species nature of the FMDV [1]. The consensus sequence of FMDV alterations occurs slowly and gradually, with approximately 0.5–1.0.5.0% of the genome undergoing modification per year, equating to around 1–2 nucleotide changes per week due to a selective advantage that helps in tracking viral spread during outbreaks [42].

Effective prevention and control of FMD is a challenging task that must be overcome through proper and effective vaccines. Therefore, the proper selection of the vaccine is the cornerstone of the restriction of the disease [13]. Bioinformatics is a crucial field that offers a wide range of applications in biological data analysis and interpretation, including evolutionary relationships, genome annotation, protein modeling, epitope prediction, and the development of novel vaccines [19]. Hence, this study focused on knowing the actual evolutionary relationship between the circulating strains to understand the situation of FMD in Egypt using different bioinformatics tools. VP1 is considered the most variable gene, boasting a 30–50% difference between serotypes and offering the key to serotype identification [24]. Interestingly, the VP1 protein harbors a highly conserved RGD (arginine-glycine-aspartic acid) motif in the G-H loop, a critical player in virus entrance through binding to host integrin receptors [41]. These receptors, a diverse family of 24 α–β heterodimers, recognize the RGD sequence in their natural ligands [43]. Moreover, the G-H loop has a hypervariable region (HV) that influences immune evasion and antibody binding [44].

According to the current study, FMDV serotype O is most common in Egypt, followed by serotype A and SAT2, and this agrees with [22]. These findings are consistent with the FMDV World Reference Laboratory, which detailed that FMDV serotype O, topotype EA-3, has dominated circulation since its first emergence in Egypt in 2012 [45].

According to the MSA of the three serotypes (A, O, and SAT2), there were mutations, most of which were synonymous. However, a few were nonsynonymous, and when accumulated year by year, they caused changes in amino acids, particularly at the immunogenic sites (G-H loop and C-terminus), leading to antigenic shifts and divergence of the serotype [46, 47]. In serotype A, a cysteine residue at position 177 in the C-terminal region plays a critical role in forming disulfide bonds. These bonds help stabilize the protein’s structure, preserving its shape and antigenic features, which can improve the match between the vaccine and circulating strains [48]. However, changes were noted in the B-C loop of serotype A, such as A51G, F56L, and A59S mutations. For instance, the shift from alanine (A) to glycine (G) at position 51 reduces the side chain size, which may increase flexibility in the loop, potentially helping the virus escape immune detection by reducing how well antibodies bind [49]. Similarly, replacing phenylalanine (F) with leucine (L) at position 56 changes an aromatic residue to an aliphatic one, which weakens hydrophobic interactions, potentially affecting the stability and visibility of the loop to antibodies [50]. Additionally, alterations in the E-F loop at position 74, where lysine (K) is replaced by glutamic acid (E), glutamine (Q), or aspartic acid (D), introduce charge shifts. Since lysine is positively charged, whereas glutamic acid and aspartic acid are negatively charged, these changes could modify the loop’s structure by causing charge-based attraction or repulsion with nearby residues, which may disrupt antibody binding and aid in immune evasion, as discussed in [51, 52].

Interestingly, the identification of a novel G136S substitution within the highly conserved RGD motif at positions 135–137 in serotype A in the Menoufia strain (MG552839) represents a significant finding. This mutation may alter the viral binding affinity to host integrins, potentially affecting viral infectivity and pathogenicity. The change from glycine to serine introduces a side chain that may modify the local structure or flexibility of the motif, potentially impacting viral entry mechanisms. To our knowledge, this is the first report of such a mutation, suggesting that it may represent an adaptive variation with implications for viral-host interactions and vaccine design targeting this motif.

In serotype O, the G-H loop remains structurally important, featuring the conserved RGD motif (positions 145–147), which is key for cell attachment. However, nearby mutations, like Q172R (glutamine to arginine), may affect local interactions. Arginine, with its positive charge, often forms salt bridges with negatively charged residues, whereas glutamine is neutral and polar, which could subtly alter the charge landscape around this region, potentially affecting cell binding or immune recognition [53, 54]. A unique mutation pattern is observed in the new topotype ON569816_Sharkia_Euro_South_America, which has changes like L36M and V78I. These modifications affect hydrophobic interactions, which may slightly alter protein folding [40]. For example, methionine (M) has a longer side chain than leucine (L) at position 36, potentially introducing new van der Waals interactions that stabilize the protein differently than the original sequence, possibly impacting vaccine efficacy [55]. In the C-terminal region, substitutions at threonine (T) position 197 (to serine (S), aspartic acid (D), glutamic acid (E), or asparagine (N)) bring varying polarity and charge changes. While serine and threonine have hydroxyl groups, they differ in size, which could influence the protein’s surface exposure and its interactions with other viral or host proteins.

In serotype SAT2, VP1 exhibits significant stability with four conserved cysteine residues at positions (74, 80, 134, and 189) that support disulfide bond formation, reinforcing robust protein folding. Nonetheless, mutations in the B-C loop, such as N45G and T48S, may impact protein function. The change from asparagine (N) to glycine (G) at position 45 removes a polar side chain, potentially disrupting hydrogen bonding with nearby residues. This increase in loop flexibility could weaken structural constraints, possibly enabling immune evasion by impairing antibody binding [56]. Additionally, while the G-H loop contains a conserved RGD motif essential for receptor binding, unique variations (like “PWG” and “SWR” motifs) in certain strains may represent region-specific adaptations. These variations may alter host-pathogen interactions that could influence viral spread and immune response. Notably, mutations observed in strains from the same province (Domyat) and different hosts underscore the virus’s adaptability across hosts and environments, which is critical from an epidemiological standpoint. Overall, the structural implications of these amino acid changes affect FMDV’s antigenicity, vaccine effectiveness, and the need for strain-specific vaccine updates. Monitoring these mutations enables improved vaccine design, as understanding how these changes influence the virus’s biochemical properties and shape can help ensure that vaccines are better matched to the circulating strains, ultimately leading to stronger immune responses in vaccinated animals.

Understanding the relationships among species through phylogenetic analysis is essential for evolutionary biology and epidemiology, enabling researchers to identify disease transmission patterns and potential outbreaks [18]. In this study, the phylogenetic analysis of the VP1 coding sequences of FMDV serotype A field strains revealed two main topotypes: Asia and Africa, consistent with previous reports [57]. Notably, our results identified the emergence of a new Europe–South America (Euro–SA) topotype in a field strain isolated from buffalo in Beheira Governorate in 2022, which caused a local outbreak. This finding is particularly significant, as previous studies neither detected this topotype in Egypt nor reported it in this governorate, indicating a recent introduction or an independent evolution event.

For serotype O, the isolates were primarily clustered within the ME-SA and EA-3 topotypes, aligning with earlier observations [23]. However, our analysis also detected a Euro–SA topotype in a field strain from Sharqia Governorate, representing the first report of this lineage in Egypt. No Euro–SA strains of serotype O had been previously described in earlier Egyptian studies, highlighting the dynamic nature of FMDV evolution and the possibility of new viral incursions from external sources.

From 2012 to 2015, the EA-3 topotype predominated, with field strains from these years aligning closely within this clade. This indicates that EA-3 strains were the primary circulating variants, which could be linked to the regional spread and introduction of strains through animal movement or trade across East African regions [58]. In 2017, however, both EA-3 and ME-SA topotypes were identified among circulating strains, highlighting a shift in epidemiological patterns. This dual presence suggests potential cross-regional introductions or co-circulation of distinct viral strains, possibly due to changes in animal movement patterns or increased international trade in livestock [59]. The circulation of ME-SA topotypes alongside EA-3 strains could pose challenges in controlling FMDV spread, as vaccines may need to be adapted to cover multiple topotypes effectively. Consequently, it is also critical to monitor the evolution of these topotypes to prevent potential vaccine mismatches due to ongoing genetic drift.

In the case of the SAT2 serotype, all the field strains were found to be related to topotype VII, which was first introduced to Egypt in 2012, consisting of two lineages: Ghb-12 and Alx-12, with Ghb-12 being the predominant one [60, 61]. Additionally, the results indicated that strains grouped into the new lineage Lib-12 emerged in 2018 [62].

The phylogenetic diversity observed among serotypes A, O, and SAT2 underscores the complex epidemiology and continuous molecular evolution of FMDV in Egypt and neighboring regions. Each serotype’s topotype distribution reflects distinct patterns of regional spread, persistence, and adaptation influenced by animal movement, trade networks, and vaccine usage. The detection of newly emerged or reintroduced topotypes, such as the Euro–SA topotype, emphasizes the urgent need for continuous molecular surveillance, timely vaccine updates, and region-specific control strategies to mitigate the spread of diverse and evolving FMDV strains.

The average evolutionary rate conducted in this study indicated that serotype SAT2 was the most variable, followed by serotypes A and O, which agreed with the findings of [63]. This variance likely reflects the temporal genetic shifts within field strains and highlights the quasi-species nature of FMDV, whereby frequent mutations lead to divergence from field strains over time. As a result, such variability underscores the need for regular monitoring updates to maintain high efficacy, especially for vaccines targeting rapidly evolving serotypes like SAT2.

The high evolutionary rate and structural stability are not mutually exclusive. The serotype’s ability to accumulate genetic changes rapidly, combined with the stabilizing effects of disulfide bonds and conservative substitutions, allows it to adapt to new challenges while maintaining its structural integrity. The analysis of substitution patterns and evolutionary divergence across the three FMDV serotypes reveals distinct stability and variability characteristics, which can inform further research and vaccine design strategies. Among the serotypes, serotype O exhibits the highest Transition/Transversion bias (*R* = 3.08), indicating a more conservative substitution pattern compared to serotype A (*R* = 2.39). This higher bias in serotype O suggests that nucleotide substitutions are more likely to be transitions, which are generally less disruptive to protein structure, thereby contributing to its relative stability. Additionally, the presence of two cysteine residues in serotype O may further contribute to its structural stability, as these residues can form disulfide bonds that help maintain protein integrity. In contrast, serotype SAT2, despite having the highest Transition/Transversion bias (*R* = 3.55), possesses four cysteine residues, which could lead to increased structural complexity and potential variability due to the formation of additional disulfide bonds. This unique combination of features makes SAT2 a particularly interesting and complex serotype for further study, especially in the context of vaccine design and understanding viral evolution.

Temporal signal strength varied among FMDV serotypes, with serotypes A and SAT2 showing moderate signals suitable for molecular clock analysis, whereas serotype O exhibited a weaker temporal signal, suggesting that additional sequence data or alternative analytical approaches may be required for more accurate time-scale estimations. The observed differences in evolutionary rates and time to the most recent common ancestor (TMRCA) likely reflect serotype-specific evolutionary pressures, population dynamics, and sampling biases. Importantly, this comprehensive evaluation of the temporal structure and evolutionary rate estimation has not been previously performed for Egyptian FMDV isolates. By integrating molecular clock modeling with phylogenetic and mutational analyses, this study provides a more complete understanding of FMDV evolution in Egypt and offers a valuable framework for future surveillance and vaccine design efforts. Future research should aim to overcome current limitations by incorporating larger, geographically diverse datasets and applying advanced evolutionary models that account for rate variation and other sources of uncertainty.

It is important to acknowledge certain limitations in this study. First, our analysis relies on sequences publicly available in the GenBank database, which may not capture all circulating field strains, and the absence of detailed geographic metadata that would allow full spatial-temporal modeling. Second, while our in silico 3D modeling of the G136S mutation provides a valuable hypothesis, experimental validation (e.g., reverse genetics) is required to confirm its true biological impact on integrin binding and viral fitness. Finally, future research should incorporate whole-genome data and structural epitope mapping to refine antigenic matching between field and vaccine strains.

## Conclusion

In conclusion, FMD is a highly contagious disease that requires careful and adaptive management. This study showed that serotype SAT2 has the highest evolutionary variability per year, followed by serotype A and then serotype O. Phylogenetic and identity matrix analyses highlighted the evolutionary relationship of field strains. Although most mutations are minor, their effects on structural conformation and immune recognition underscore the need to monitor these mutations, especially within key antigenic regions. This is essential to ensure continued vaccine efficacy against evolving field strains and to refine vaccine design. Analyzing these mutations’ biochemical and conformational effects can improve strain matching and enhance immune responses in vaccinated populations. Additionally, the phylogenetic diversity seen in serotypes A, O, and SAT2 highlights the complex epidemiology of FMDV in Egypt and neighboring regions, affected by animal movement, trade, and the implementation of vaccines. The emergence of new topotypes or re-introductions of rare lineages emphasizes the need for regular surveillance and region-specific strategies for timely vaccine updates and disease control to prevent the spread of diverse FMDV strains.

## Supplementary Information

Supplementary Material 1.

## Data Availability

All data generated or analyzed during this study are included in this published article and its supplementary information files.

## Abbreviations

FMD: Foot and Mouth Disease
FMDV: Foot and Mouth Disease Virus
VP1: Viral Protein 1
MEVAC: Middle East for Vaccines
ME-SA: Middle East-South Asia
EA-3: East Africa-3
NCBI: National Center of Biotechnology Institute
MSA: Multiple Sequence Alignment
JTT: Jones-Taylor-Thornton
MCMC: Markov Chain Monte Carlo
ESS: Effective sample size

## References

[1] Brito BP, Rodriguez LL, Hammond JM, Pinto J, Perez AM. Review of the Global Distribution of Foot-and-Mouth Disease Virus from 2007 to 2014. Transbound Emerg Dis [Internet]. 2017;64:316–32. Available from: https://pubmed.ncbi.nlm.nih.gov/25996568/10.1111/tbed.12373 25996568

[2] Grubman MJ, Baxt B. Foot-and-mouth disease. Clin Microbiol Rev [Internet]. 2004;17:465–93. Available from: https://pubmed.ncbi.nlm.nih.gov/15084510/10.1128/CMR.17.2.465-493.2004 PMC387408 15084510

[3] Aslam M, Alkheraije KAA. The prevalence of foot-and-mouth disease in Asia. Front Vet Sci. 2023. 10.3389/fvets.2023.1201578. 37456961 10.3389/fvets.2023.1201578 PMC10347409

[4] Arzt J, Juleff N, Zhang Z, Rodriguez LL. The pathogenesis of foot-and-mouth disease I: viral pathways in cattle. Transbound Emerg Dis [Internet]. 2011;58:291–304. Available from: https://pubmed.ncbi.nlm.nih.gov/21366894/10.1111/j.1865-1682.2011.01204.x 21366894

[5] Jamal SM, Belsham GJ. Foot-and-mouth disease: Past, present and future. Vet Res [Internet]. 2013;44:1–14. Available from: https://veterinaryresearch.biomedcentral.com/articles/10.1186/1297-9716-44-116 10.1186/1297-9716-44-116 PMC4028749 24308718

[6] Elrashedy A, Nayel M, Salama A, Zaghawa A, El-Shabasy RM, Hasan ME. Foot-and-mouth disease: genomic and proteomic structure, antigenic sites, serotype relationships, immune evasion, recent vaccine development strategies, and future perspectives. Vet Res. 2025. 10.1186/s13567-025-01485-0. 40197411 10.1186/s13567-025-01485-0 PMC11974090

[7] Hassan AM, Zaher MR, Hassanien RT, Abd-El-Moniem MI, Habashi AR, Ibraheem EM, et al. Molecular detection, phylogenetic analysis and genetic diversity of recently isolated foot-and-mouth disease virus serotype A African topotype, genotype IV. Virol J. 2022;19:1–9. 34980196 10.1186/s12985-021-01693-y PMC8722054

[8] Abu-Elnaga HI, Rizk SA, Daoud HM, Mohamed AA, Mossad W, Gamil MA, et al. Comparative nucleotide sequencing of the VP1 capsid gene of recent isolates of foot-and-mouth disease virus serotype O from Egypt. Arch Virol. 2020;165:2021–8. 10.1007/s00705-020-04708-1. 32601957 10.1007/s00705-020-04708-1

[9] Rahman SA, El, Hoffmann B, Karam R, El-Beskawy M, Hamed MF, Forth LF et al. Sequence Analysis of Egyptian Foot-and-Mouth Disease Virus Field and Vaccine Strains: Intertypic Recombination and Evidence for Accidental Release of Virulent Virus. Viruses [Internet]. 2020;12:990. Available from: https://www.mdpi.com/1999-4915/12/9/990 10.3390/v12090990 PMC7552000 32899903

[10] ِbd El-Rhman MM, Salem SA, Bazid A-HI, Abo El-Hassan DJ. Molecular and serological typing of foot-and-mouth disease virus serotypes currently circulating in Egypt. Iraqi J Vet Sci [Internet]. 2021;35:581–8. Available from: https://vetmedmosul.com/article_168010.html

[11] Hosny WAEW, Baheeg EM, Raheem Aly HA, El AE, Nabi SS, Hanna NM. Field serological investigation for peste des petits ruminants, foot-and-mouth disease, and bluetongue diseases in illegally introduced animals in Egypt. Vet World. 2020;13:1661–6. 33061242 10.14202/vetworld.2020.1661-1666 PMC7522941

[12] Belsham GJ. Towards improvements in foot-and-mouth disease vaccine performance. Acta Vet Scand [Internet]. 2020;62. Available from: https://pubmed.ncbi.nlm.nih.gov/32434544/10.1186/s13028-020-00519-1 PMC7240906 32434544

[13] Kenubih A. Foot and Mouth Disease Vaccine Development and Challenges in Inducing Long-Lasting Immunity: Trends and Current Perspectives. Vet Med Res Reports [Internet]. 2021;12:205. Available from: /pmc/articles/PMC8420785/10.2147/VMRR.S319761 PMC8420785 34513635

[14] Lu Z, Yu S, Wang W, Chen W, Wang X, Wu K, et al. Development of foot-and-mouth disease vaccines in recent years. VACCINES. 2022. 10.3390/vaccines10111817. 36366327 10.3390/vaccines10111817 PMC9693445

[15] Elrashedy A, Mousa W, Nayel M, Salama A, Zaghawa A, Elsify A, et al. Systematic review and meta-analysis of the effectiveness of polypeptide, virus-like particles, and viral vector vaccines for foot-and-mouth disease (2020–2025). Sci Rep. 2025. 10.1038/s41598-025-24078-5. 41214185 10.1038/s41598-025-24078-5 PMC12603288

[16] Jamal SM, Shirazi MHN, Ozyoruk F, Parlak U, Normann P, Belsham GJ. Evidence for multiple recombination events within foot-and-mouth disease viruses circulating in West Eurasia. Transbound Emerg Dis [Internet]. 2020;67:979–93. Available from: https://pubmed.ncbi.nlm.nih.gov/31758840/10.1111/tbed.13433 31758840

[17] Elrashedy A, Mousa W, Nayel M, Salama A, Zaghawa A, Elsify A et al. Advances in bioinformatics and multi-omics integration: transforming viral infectious disease research in veterinary medicine. Virol J [Internet]. 2025;22:22. Available from: https://virologyj.biomedcentral.com/articles/10.1186/s12985-025-02640-x 10.1186/s12985-025-02640-x PMC11783962 39891257

[18] Elrashedy A, Nayel M, Salama A, Zaghawa A, Abdelsalam NR. Phylogenetic analysis and comparative genomics of *Brucella abortus* and *Brucella melitensis* strains in Egypt. J Mol Evol. 2024. 10.1007/s00239-024-10173-0. 38809331 10.1007/s00239-024-10173-0 PMC11169049

[19] Elrashedy A, Nayel M, Salama A, Hasan ME. Applications of bioinformatics on genomics and proteomics levels highlighted in Brucella pathogen. J Curr Vet Res. 2024;6:1–15.

[20] Brito B, Pauszek SJ, Eschbaumer M, Stenfeldt C, de Carvalho Ferreira HC, Vu LT et al. Phylodynamics of foot-and-mouth disease virus O/PanAsia in Vietnam 2010–2014. Vet Res [Internet]. 2017;48:24. Available from: https://veterinaryresearch.biomedcentral.com/articles/10.1186/s13567-017-0424-7 10.1186/s13567-017-0424-7 PMC5390394 28403902

[21] Elrashedy A, Nayel M, Salama A, Salama MM, Hasan ME. Bioinformatics approach for structure modeling, vaccine design, and molecular docking of Brucella candidate proteins BvrR, OMP25, and OMP31. Sci Rep. 2024;14:11951. 38789443 10.1038/s41598-024-61991-7 PMC11126717

[22] Hassanein RT, Abdelmegeed HK, Abdelwahed DA, Zaki AG, Saad AS, Shahein MA et al. Epidemiological and Genetic Insights of the Circulating Foot-and-Mouth Disease Virus Serotypes in Egypt. Curr Microbiol [Internet]. 2024;81:435. Available from: https://link.springer.com/10.1007/s00284-024-03944-x 10.1007/s00284-024-03944-x PMC11525254 39477883

[23] Hassan A, Aboezz Z, El-Habbaa A, Shahein M, Hagag N, Sharawy S. Phylogenetic analysis of VP1 foot and mouth disease virus strains circulating between 2018–2020, Egypt. Benha Vet Med J [Internet]. 2022;90–6. Available from: https://bvmj.journals.ekb.eg/article_268435.html

[24] Woldemariyam F, Paeshuyse J. Viral Protein 1 (VP1) Sequence-Based Genetic Diversity of SAT 2 FMDV Circulating in Ethiopia from 1990 to 2015. Vet Med Res Reports [Internet]. 2023;Volume 14:91–101. Available from: https://www.dovepress.com/viral-protein-1-vp1-sequence-based-genetic-diversity-of-sat-2-fmdv-cir-peer-reviewed-fulltext-article-VMRR 10.2147/VMRR.S408352 PMC10226516 37256222

[25] Thompson JD, Higgins DG, Gibson TJ. CLUSTAL W: improving the sensitivity of progressive multiple sequence alignment through sequence weighting, position-specific gap penalties and weight matrix choice. Nucleic Acids Res [Internet]. 1994;22:4673–80. Available from: https://academic.oup.com/nar/article-lookup/doi/10.1093/nar/22.22.4673 10.1093/nar/22.22.4673 PMC308517 7984417

[26] Muhire BM, Varsani A, Martin DP. SDT: A Virus Classification Tool Based on Pairwise Sequence Alignment and Identity Calculation. Kuhn JH, editor. PLoS One [Internet]. 2014;9:e108277. Available from: 10.1371/journal.pone.0108277 10.1371/journal.pone.0108277 PMC4178126 25259891

[27] Edgar RC. MUSCLE: multiple sequence alignment with high accuracy and high throughput. Nucleic Acids Res [Internet]. 2004;32:1792–7. Available from: https://academic.oup.com/nar/article-lookup/doi/10.1093/nar/gkh340 10.1093/nar/gkh340 PMC390337 15034147

[28] Nei M, Kumar S. Molecular evolution and phylogenetics. Oxford Univ. Press; 2000.

[29] Tamura K, Nei M. Estimation of the number of nucleotide substitutions in the control region of mitochondrial DNA in humans and chimpanzees. Mol Biol Evol [Internet]. 1993; Available from: https://academic.oup.com/mbe/article/10/3/512/1016366/Estimation-of-the-number-of-nucleotide 10.1093/oxfordjournals.molbev.a040023 8336541

[30] Jones DT, Taylor WR, Thornton JM. The rapid generation of mutation data matrices from protein sequences. Bioinformatics [Internet]. 1992;8:275–82. Available from: https://academic.oup.com/bioinformatics/article-lookup/doi/10.1093/bioinformatics/8.3.275 10.1093/bioinformatics/8.3.275 1633570

[31] Tamura K, Stecher G, Kumar S. MEGA11: Molecular Evolutionary Genetics Analysis Version 11. Battistuzzi FU, editor. Mol Biol Evol [Internet]. 2021;38:3022–7. Available from: https://academic.oup.com/mbe/article/38/7/3022/6248099 10.1093/molbev/msab120 PMC8233496 33892491

[32] Rambaut A, Lam TT, Max Carvalho L, Pybus OG. Exploring the temporal structure of heterochronous sequences using TempEst (formerly Path-O-Gen). Virus Evol [Internet]. 2016;2:vew007. Available from: https://academic.oup.com/ve/article-lookup/doi/10.1093/ve/vew007 10.1093/ve/vew007 PMC4989882 27774300

[33] Gill MS, Lemey P, Suchard MA, Rambaut A, Baele G. Online Bayesian Phylodynamic Inference in BEAST with Application to Epidemic Reconstruction. Rosenberg M, editor. Mol Biol Evol [Internet]. 2020;37:1832–42. Available from: https://academic.oup.com/mbe/article/37/6/1832/5758268 10.1093/molbev/msaa047 PMC7253210 32101295

[34] Drummond AJ, Ho SYW, Phillips MJ, Rambaut A. Relaxed Phylogenetics and Dating with Confidence. Penny D, editor. PLoS Biol [Internet]. 2006;4:e88. Available from: https://dx.plos.org/10.1371/journal.pbio.0040088 10.1371/journal.pbio.0040088 PMC1395354 16683862

[35] Kingman JFC. The coalescent. Stoch Process their Appl [Internet]. 1982;13:235–48. Available from: https://linkinghub.elsevier.com/retrieve/pii/0304414982900114

[36] Sammut C. Markov Chain Monte Carlo. Encycl Mach Learn [Internet]., Boston MA. Springer US; 2011. pp. 639–42. Available from: https://link.springer.com/10.1007/978-0-387-30164-8_511

[37] Rambaut A, Drummond AJ, Xie D, Baele G, Suchard MA. Posterior Summarization in Bayesian Phylogenetics Using Tracer 1.7. Susko E, editor. Syst Biol [Internet]. 2018;67:901–4. Available from: https://academic.oup.com/sysbio/article/67/5/901/4989127 10.1093/sysbio/syy032 PMC6101584 29718447

[38] Yang Z, Zeng X, Zhao Y, Chen R. AlphaFold2 and its applications in the fields of biology and medicine. Signal Transduct Target Ther [Internet]. 2023;8:115. Available from: https://www.nature.com/articles/s41392-023-01381-z 10.1038/s41392-023-01381-z PMC10011802 36918529

[39] Fry EE. Structure of Foot-and-mouth disease virus serotype A1061 alone and complexed with oligosaccharide receptor: receptor conservation in the face of antigenic variation. J Gen Virol [Internet]. 2005;86:1909–20. Available from: https://www.microbiologyresearch.org/content/journal/jgv/10.1099/vir.0.80730-0 10.1099/vir.0.80730-0 15958669

[40] Mushtaq H, Shah SS, Zarlashat Y, Iqbal M, Abbas W. Cell Culture Adaptive Amino Acid Substitutions in FMDV Structural Proteins: A Key Mechanism for Altered Receptor Tropism. Viruses [Internet]. 2024;16:512. Available from: https://www.mdpi.com/1999-4915/16/4/512 10.3390/v16040512 PMC11054764 38675855

[41] Wang G, Wang Y, Shang Y, Zhang Z, Liu X. How foot-and-mouth disease virus receptor mediates foot-and-mouth disease virus infection. Virol J [Internet]. 2015;12:9. Available from: https://virologyj.biomedcentral.com/articles/10.1186/s12985-015-0246-z 10.1186/s12985-015-0246-z PMC4322448 25645358

[42] Agol VI, Gmyl AP. Emergency Services of Viral RNAs: Repair and Remodeling. Microbiol Mol Biol Rev [Internet]. 2018;82. Available from: https://journals.asm.org/doi/10.1128/MMBR.00067-17 10.1128/MMBR.00067-17 PMC5968460 29540453

[43] Ludwig BS, Kessler H, Kossatz S, Reuning U. RGD-Binding Integrins Revisited: How Recently Discovered Functions and Novel Synthetic Ligands (Re-)Shape an Ever-Evolving Field. Cancers (Basel) [Internet]. 2021;13:1711. Available from: https://www.mdpi.com/2072-6694/13/7/1711 10.3390/cancers13071711 PMC8038522 33916607

[44] Hussein HAM, Walker LR, Abdel-Raouf UM, Desouky SA, Montasser AKM, Akula SM. Beyond RGD: virus interactions with integrins. Arch Virol 2015 16011 [Internet]. 2015;160:2669–81. Available from: https://link.springer.com/article/10.1007/s00705-015-2579-8 10.1007/s00705-015-2579-8 PMC7086847 26321473

[45] Kandeil A, El-Shesheny R, Kayali G, Moatasim Y, Bagato O, Darwish M et al. Characterization of the recent outbreak of foot-and-mouth disease virus serotype SAT2 in Egypt. Arch Virol [Internet]. 2013;158:619–27. Available from: https://pubmed.ncbi.nlm.nih.gov/23132412/10.1007/s00705-012-1529-y 23132412

[46] Al-Ebshahy E, El-Ansary RE, Zhang J, Badr Y, Rady A, El-Ashram S et al. Sequence and phylogenetic analysis of FMD virus isolated from two outbreaks in Egypt. Infect Genet Evol [Internet]. 2024;123:105651. Available from: https://linkinghub.elsevier.com/retrieve/pii/S1567134824001023 10.1016/j.meegid.2024.105651 39089501

[47] Li Q, Wubshet AK, Wang Y, Heath L, Zhang J. B and T Cell Epitopes of the Incursionary Foot-and-Mouth Disease Virus Serotype SAT2 for Vaccine Development. Viruses [Internet]. 2023;15:797. Available from: https://www.mdpi.com/1999-4915/15/3/797 10.3390/v15030797 PMC10059872 36992505

[48] Wiedemann C, Kumar A, Lang A, Ohlenschläger O. Cysteines and Disulfide Bonds as Structure-Forming Units: Insights From Different Domains of Life and the Potential for Characterization by NMR. Front Chem [Internet]. 2020;8. Available from: https://www.frontiersin.org/article/10.3389/fchem.2020.00280/full 10.3389/fchem.2020.00280 PMC7191308 32391319

[49] Zhang Z, Zhang J, Wang J. Surface charge changes in spike RBD mutations of SARS-CoV-2 and its variant strains alter the virus evasiveness via HSPGs: A review and mechanistic hypothesis. Front Public Heal [Internet]. 2022;10. Available from: https://www.frontiersin.org/articles/10.3389/fpubh.2022.952916/full 10.3389/fpubh.2022.952916 PMC9449321 36091499

[50] Galles GD, Infield DT, Clark CJ, Hemshorn ML, Manikandan S, Fazan F et al. Tuning phenylalanine fluorination to assess aromatic contributions to protein function and stability in cells. Nat Commun [Internet]. 2023;14:59. Available from: https://www.nature.com/articles/s41467-022-35761-w 10.1038/s41467-022-35761-w PMC9813137 36599844

[51] Caridi F, López-Argüello S, Rodríguez-Huete A, Torres E, Bustos MJ, Cañas-Arranz R et al. Negatively charged amino acids at the foot-and-mouth disease virus capsid reduce the virion-destabilizing effect of viral RNA at acidic pH. Sci Rep [Internet]. 2020;10:1657. Available from: https://www.nature.com/articles/s41598-020-58414-8 10.1038/s41598-020-58414-8 PMC6997383 32015411

[52] Hossain KA, Anjume H, Alam KMM, Yeamin A, Akter S, Hossain MA, et al. Emergence of a novel sublineage, MYMBD21 under SA-2018 lineage of foot-and-mouth disease virus serotype O in Bangladesh. Sci Rep. 2023;13:1–12. 10.1038/s41598-023-36830-w. 37330573 10.1038/s41598-023-36830-w PMC10276842

[53] Caridi F, Cañas-Arranz R, Vázquez-Calvo Á, de León P, Calderón KI, Domingo E, et al. Adaptive value of foot-and-mouth disease virus capsid substitutions with opposite effects on particle acid stability. Sci Rep. 2021;11:1–11. 10.1038/s41598-021-02757-3. 34873184 10.1038/s41598-021-02757-3 PMC8648728

[54] Fuhrmann J, Clancy KW, Thompson PR. Chemical Biology of Protein Arginine Modifications in Epigenetic Regulation. Chem Rev [Internet]. 2015;115:5413–61. Available from: https://pubs.acs.org/doi/10.1021/acs.chemrev.5b00003 10.1021/acs.chemrev.5b00003 PMC4463550 25970731

[55] Gaur A, Lipponen J, Yang Y, Armen RS, Wang B. Mutation of Methionine to Asparagine but Not Leucine in Parathyroid Hormone Mimics the Loss of Biological Function upon Oxidation. Biochemistry [Internet]. 2022;61:981–91. Available from: https://pubs.acs.org/doi/10.1021/acs.biochem.2c00200 10.1021/acs.biochem.2c00200 PMC9179810 35533300

[56] Soriano-Correa C, Barrientos-Salcedo C, Campos-Fernández L, Alvarado-Salazar A, Esquivel RO. Importance of asparagine on the conformational stability and chemical reactivity of selected anti-inflammatory peptides. Chem Phys [Internet]. 2015;457:180–7. Available from: https://linkinghub.elsevier.com/retrieve/pii/S0301010415001792

[57] Shahein MA, Hussein HA, Ali MH, Ghoniem SM, Shemies OA, Afify AF et al. Circulating foot-and-mouth disease virus serotype A African-genotype IV in Egypt during 2022. Vet World [Internet]. 2023;1429–37. Available from: https://www.veterinaryworld.org/Vol.16/July-2023/8.html 10.14202/vetworld.2023.1429-1437 PMC10446720 37621542

[58] Metwally S, Bkear N, Badr Y, Elshafey B, Alhag SK, Al-Shuraym LA et al. A Newly Emerging Serotype A Strain in Foot-and-Mouth Disease Virus with Higher Severity and Mortality in Buffalo than in Cattle Calves in North Egypt. Vet Sci [Internet]. 2023;10:488. Available from: https://www.mdpi.com/2306-7381/10/8/488 10.3390/vetsci10080488 PMC10457878 37624275

[59] Bertram MR, de Rueda CB, Garabed R, Jumbo SD, Moritz M, Pauszek S, et al. Molecular epidemiology of foot-and-mouth disease virus in the context of transboundary animal movement in the Far North region of Cameroon. Front Vet Sci. 2018;5:1–14. 30619901 10.3389/fvets.2018.00320 PMC6301994

[60] Ahmed HA, Salem SAH, Habashi AR, Arafa AA, Aggour MGA, Salem GH et al. Emergence of Foot-and-Mouth Disease Virus SAT 2 in Egypt During 2012. Transbound Emerg Dis [Internet]. 2012;59:476–81. Available from: https://onlinelibrary.wiley.com/doi/10.1111/tbed.12015 10.1111/tbed.12015 23025522

[61] Hassan AM, El-mayet FS, El-Habbaa AS, Shahein MA, El Zowalaty ME, Hagag NM, et al. Molecular characterization of newly emerging foot-and-mouth disease virus serotype SAT 2 of Lib-12 lineage isolated from Egypt. Virus Res. 2022;311:198651. 10.1016/j.virusres.2021.198651. 34879242 10.1016/j.virusres.2021.198651

[62] Soltan MA, Dohreig RMA, Abbas H, Ellawa M, Yousif I, Aly AE et al. Emergence of foot and mouth disease virus, Lib-12 lineage of topotype < scp > VII, serotype < scp > SAT 2 in Egypt, 2018. Transbound Emerg Dis [Internet]. 2019;66:1105–6. Available from: https://onlinelibrary.wiley.com/doi/10.1111/tbed.13152 10.1111/tbed.13152 30779326

[63] Nahas AF, El, Salem SAH. Meta-analysis of genetic diversity of the VP1 gene among the circulating O, A, and SAT2 serotypes and vaccine strains of FMD virus in Egypt. J Vet Res [Internet]. 2020;64:487–93. Available from: https://www.sciendo.com/article/10.2478/jvetres-2020-0069 10.2478/jvetres-2020-0069 PMC7734679 33367136

