# Supplementary material for: Phylogenetic and evolutionary analysis of VP1 coding sequences of foot-and-mouth disease virus serotypes A, O, and SAT2 in Egypt
Source: Virol J. 2025 Dec 27;23:24. doi: 10.1186/s12985-025-03039-4 (PMC12849381; doi:10.1186/s12985-025-03039-4)
Supplement: Supplementary file 1 — Supplementary Material 1. [file 12985_2025_3039_MOESM1_ESM.docx]

**Table (S1) The total sequences retrieved of A, O, and SAT2 serotypes of FMDV in Egypt**

| Serotype A | | | | | | | | | |
| --- | --- | --- | --- | --- | --- | --- | --- | --- | --- |
| MW792216 | MW792217 | OL769314 | OL769315 | MT597128 | MT508912 | MT442149 | KC888938 | KC888939 | MT442154 |
| KR092701 | MT442146 | MH732982 | MT863265 | MG552838 | MG552842 | MT442153 | MG552841 | MG552839 | EF208773 |
| MH732981 | MN853329 | EF208756 | EF208757 | EF208762 | EF208765 | EF208766 | EF208774 | EF208769 | MK422570 |
| KX083565 | KT699210 | MG552840 | MT597129 | MT863267 | MT199285 | MG957500 | KC888937 | MG552837 | MN853330 |
| KX447000 | MT863264 | MT863266 | MT442165 | MT442167 | ON380439 | ON380440 | OL456140 | OP321262 | KX446998 |
| OQ302221 | OQ302225 | MT597127 | MG552843 | EF208770 | OP823161 | MK572799 | MK422573 | MK422574 |  |
| Serotype O | | | | | | | | | |
| GU566059 | KX258001 | MT443080 | OM221178 | OM221189 | KJ210073 | AJ251477 | MF322684 | ON569816 | JQ837834 |
| KT121465 | KX258004 | MN296504 | GU566059 | OM221191 | OM221201 | OM221233 | MF322685 | MF962876 | JQ837835 |
| EU553840 | MG552844 | MN296503 | MG552846 | MG552845 | MN296506 | OM221183 | OM221196 | MT597125 | OM221194 |
| MG552848 | OM221212 | MF322680 | OM221185 | MN296507 | OM221204 | OM221205 | OM221232 | MT443078 | OM221186 |
| MG571528 | OM221197 | OM681355 | MT597122 | MT597123 | KT121467 | MT597124 | MF322683 | MF322679 | MG552849 |
| Serotype SAT 2 | | | | | | | | | |
| JX570617 | JX570633 | KC440884 | MZ097483 | JX570622 | MT597121 | MT597120 | MZ097480 | MZ097482 | MG552850 |
| JX013980 | KX258067 | MT199283 | MZ097481 | KY372408 | KY372409 | KY372410 | MT450473 | MT597118 | MT597119 |
| JX570620 | KX258066 | MF322695 | MZ146910 | MH732988 | MH732987 | MH732985 | MH732984 | MZ097479 | JX014255 |
| JX570621 | KF112932 | MF322696 | OL448982 | KF112936 | KF112937 | KF055860 | KF055861 | KX258063 | KX258064 |
| KF112935 | KF112934 | JX570623 | JX570624 | JX570625 | JX570626 | MN864516 | JX570627 | MG552851 | MT602089 |


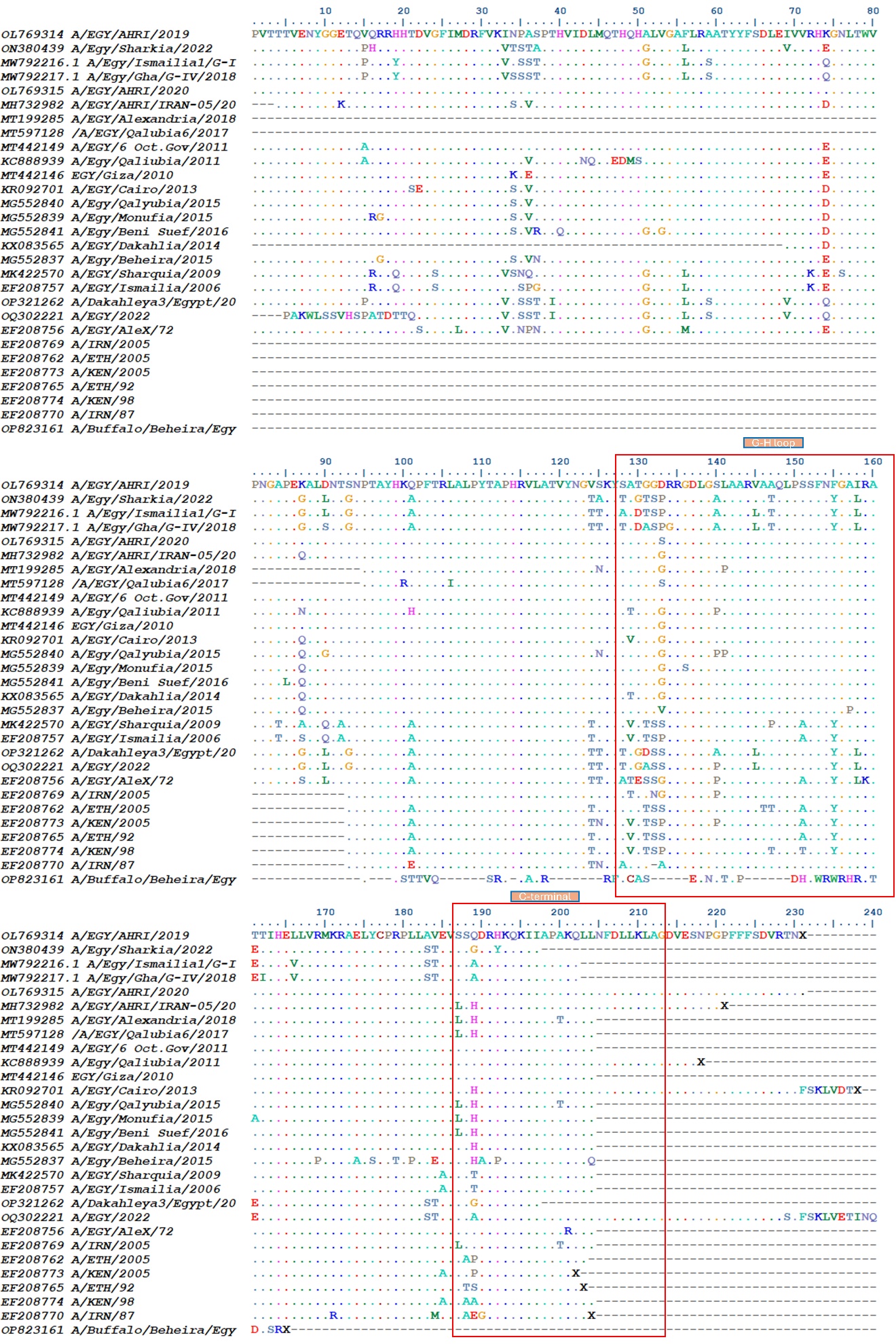


Fig. S1 Multiple sequence alignment of the VP1 protein of FMDV of serotype A.


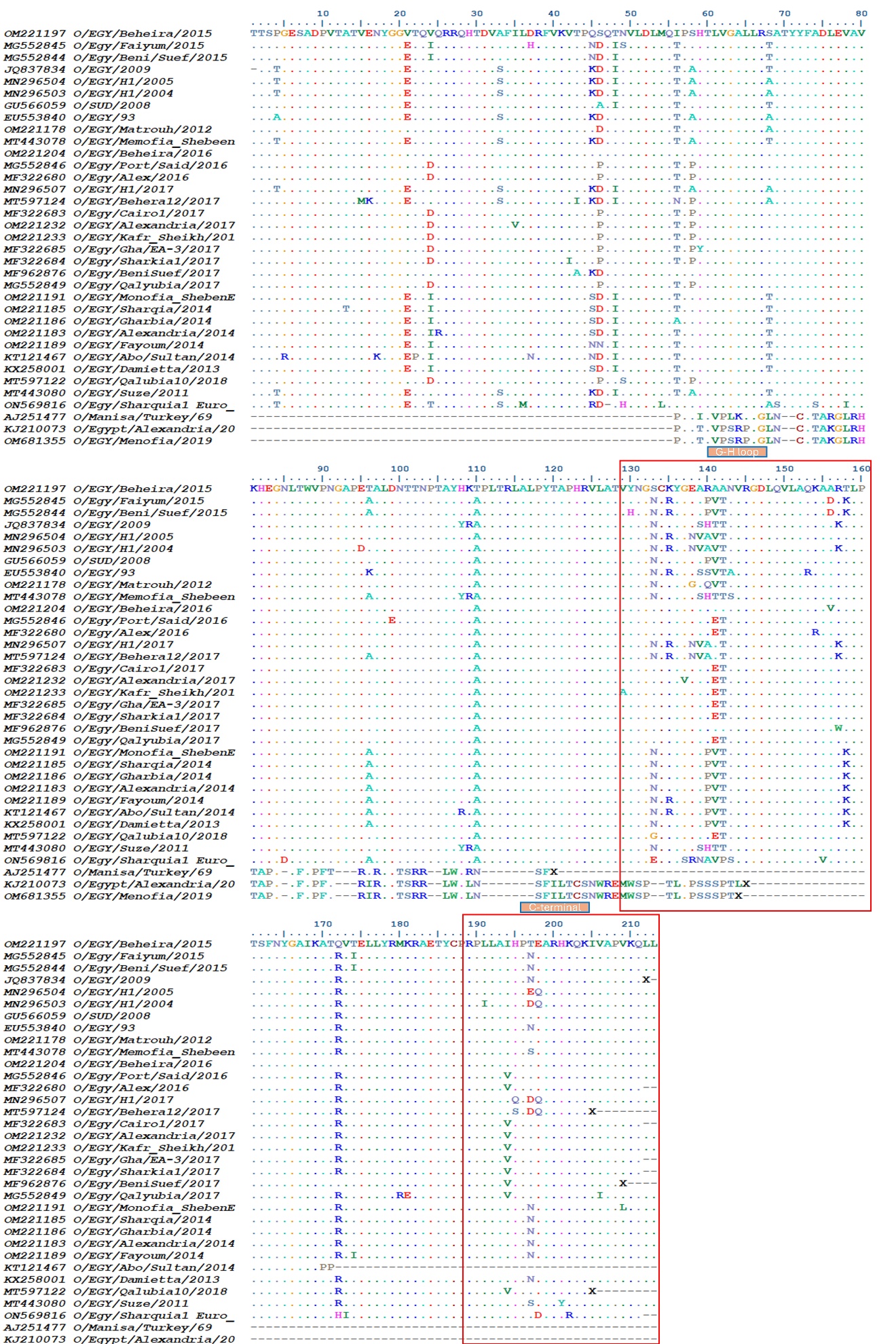


Fig. S2 Multiple sequence alignment of the VP1 protein of FMDV of serotype O.


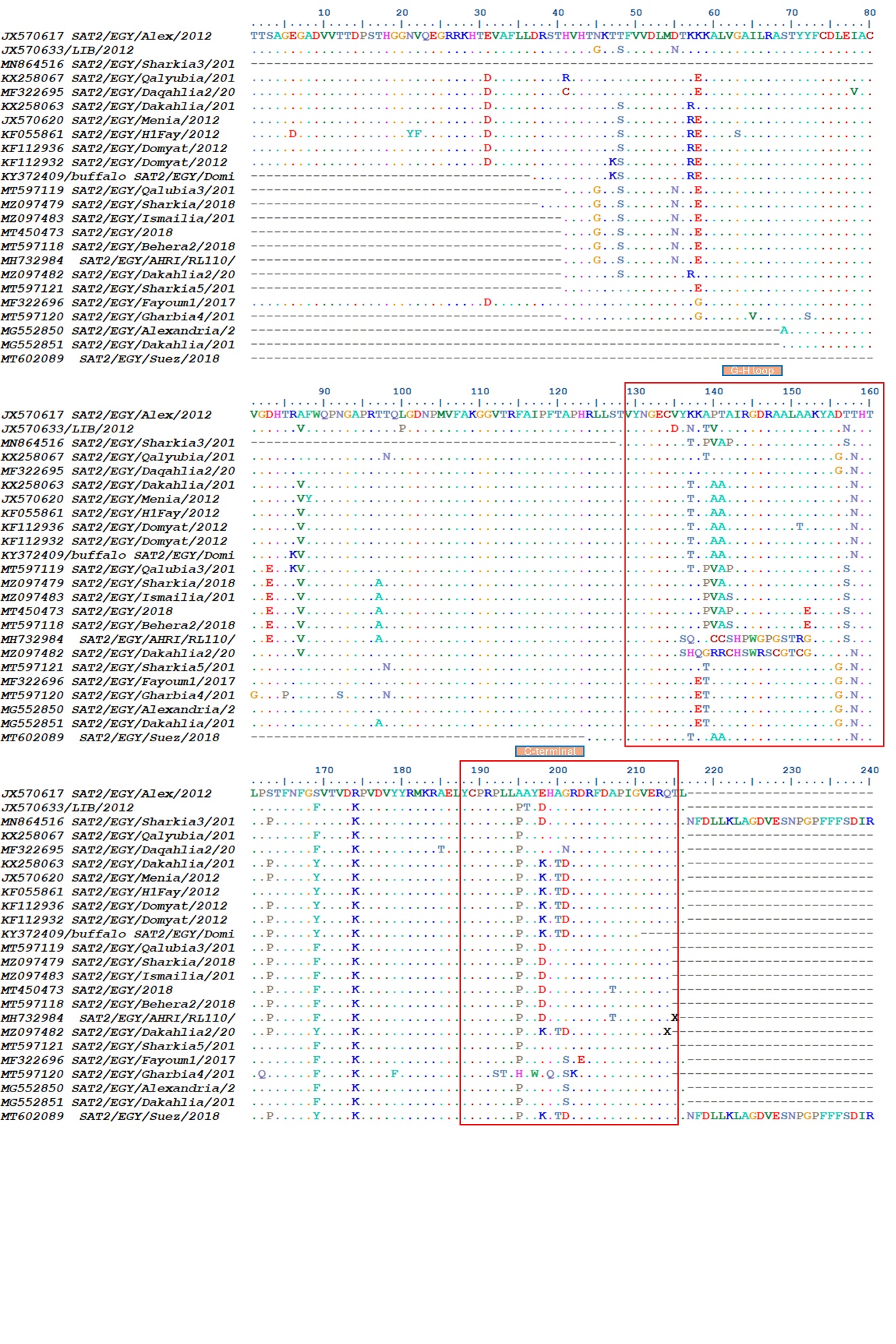


Fig. S3 Multiple sequence alignment of the VP1 of FMDV of serotype SAT2.


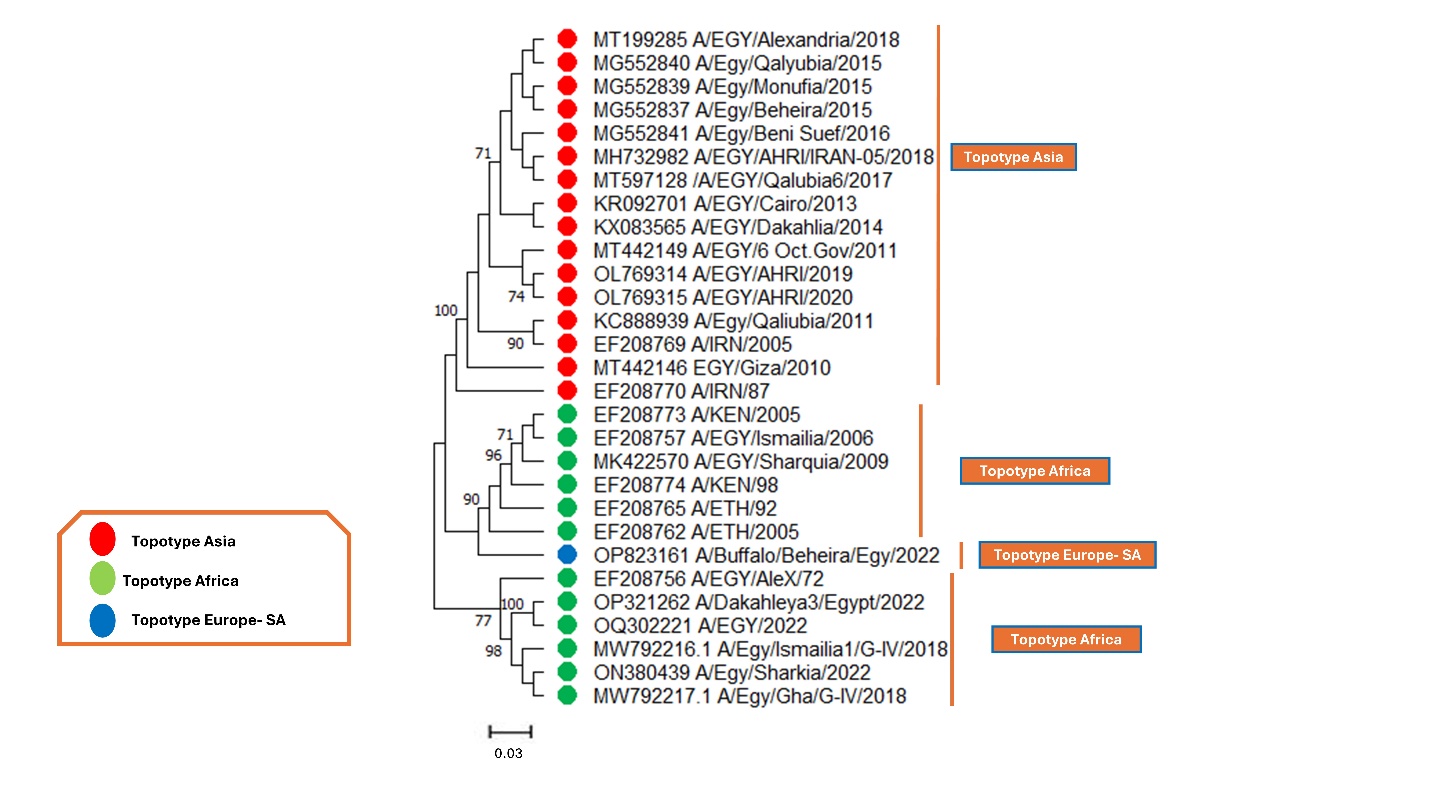


Fig. S4: The evolutionary history was inferred using the Maximum Likelihood method and the JTT model of serotype A. The percentage of trees in which the associated taxa clustered together is shown next to the branches. Initial tree(s) for the heuristic search were obtained automatically by applying Neighbor-Join and BioNJ algorithms to a matrix of pairwise distances estimated using the JTT model and then selecting the topology with superior log likelihood value. The coding data was translated assuming a Standard genetic code table. This analysis involved 29 protein sequences of serotype A. The red circles pointed to Asia, the green circles referred to Africa, and the blue circles indicated Europe-SA.


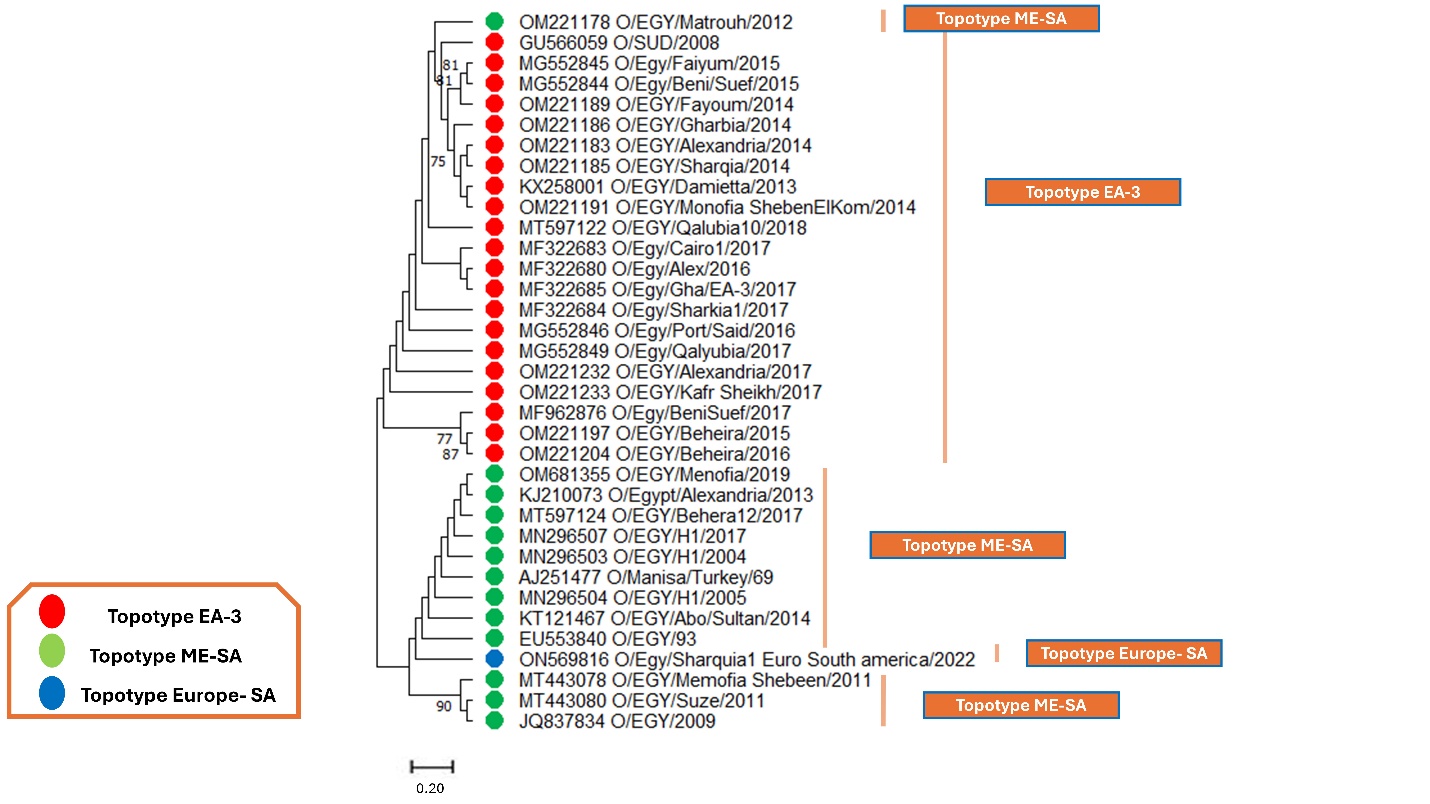


Fig. S5: The evolutionary history was inferred using the Maximum Likelihood method and the JTT model of serotype O. This analysis involved 42 protein sequences of serotype O. The percentage of trees in which the associated taxa clustered together is shown next to the branches. Initial tree(s) for the heuristic search were obtained automatically by applying Neighbor-Join and BioNJ algorithms to a matrix of pairwise distances estimated using the JTT model and then selecting the topology with superior log likelihood value. This analysis involved 35 protein sequences of serotype SAT2. The coding data was translated assuming a Standard genetic code table. The red circles pointed to East Africa-3 (EA-3), the green circles referred to the Middle East-South Asia (ME-SA), and the blue circles indicated Europe-SA.


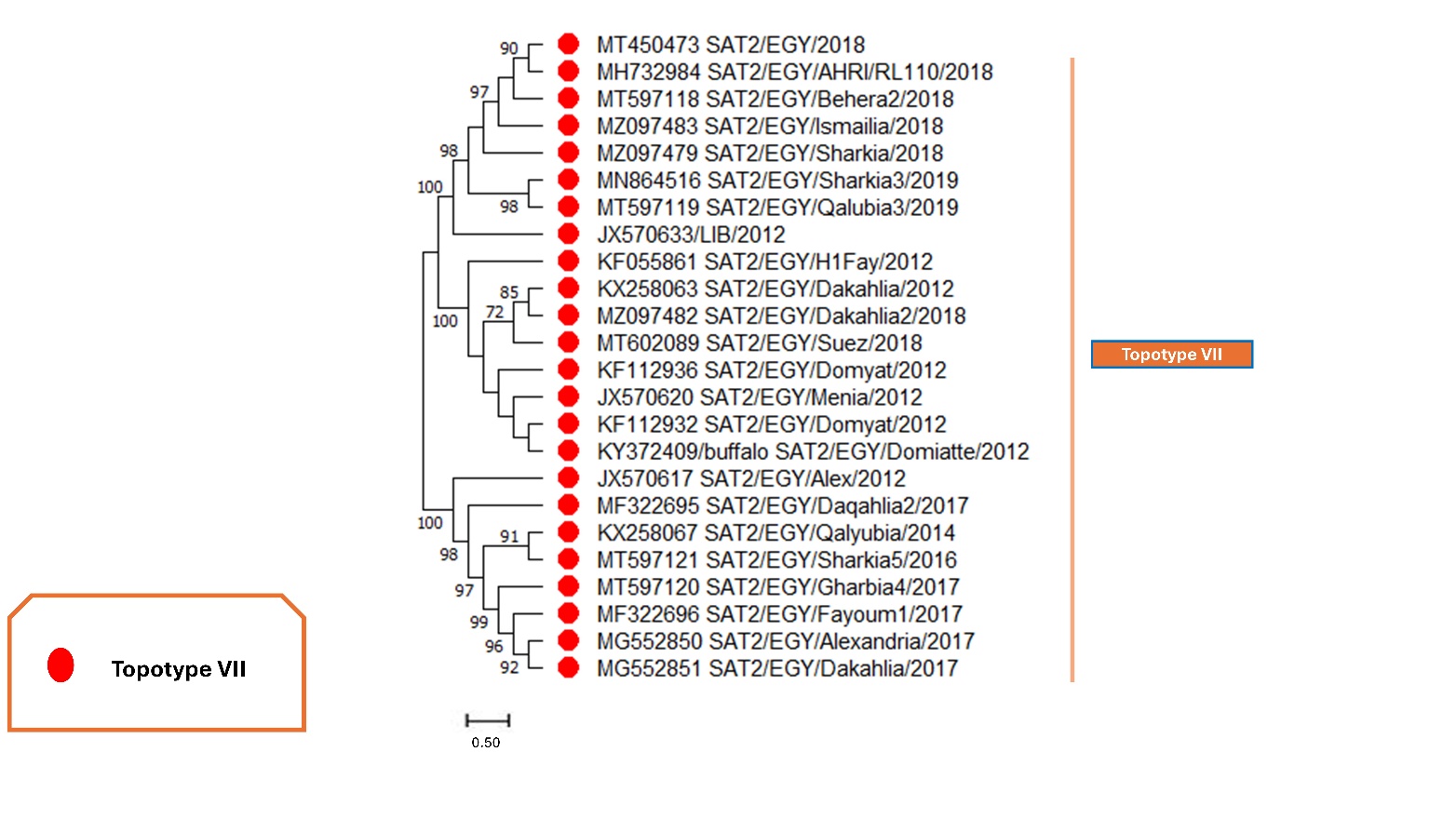


Fig. S6: The evolutionary history was inferred using the Maximum Likelihood method and the JTT model of serotype SAT2. The percentage of trees in which the associated taxa clustered together is shown next to the branches. Initial tree(s) for the heuristic search were obtained automatically by applying Neighbor-Join and BioNJ algorithms to a matrix of pairwise distances estimated using the JTT model and then selecting the topology with superior log likelihood value. This analysis involved 24 protein sequences of serotype SAT2. The coding data was translated assuming a Standard genetic code table. The red circles pointed to topotype VII.
